# Opposing roles for myeloid and smooth muscle cell STING in pulmonary hypertension

**DOI:** 10.1172/jci.insight.184792

**Published:** 2025-05-22

**Authors:** Ann T. Pham, Shiza Virk, Aline C. Oliveira, Matthew D. Alves, Chunhua Fu, Yutao Zhang, Jimena Alvarez-Castanon, Brian B. Lee, Keira L. Lee, Radwan Mashina, Katherine E. Ray, Patrick Donabedian, Elnaz Ebrahimi, Harsh Patel, Reeha Patel, Duncan Lewis, Zhiguang Huo, Harry Karmouty-Quintana, Li Chen, Lei Jin, Andrew J. Bryant

**Affiliations:** 1 Department of Medicine,; 2 Department of Pharmacology and Therapeutics, and; 3 Department of Biostatistics, College of Medicine, University of Florida, Gainesville, Florida, USA.; 4 Department of Biochemistry and Molecular Biology, Division of Pulmonary, Critical Care, and Sleep Medicine, McGovern Medical School, University of Texas Health Science Center at Houston, Houston, Texas, USA.

**Keywords:** Pulmonology, Vascular biology, Cardiovascular disease

## Abstract

There is an emerging role for stimulator of interferon genes (STING) signaling in pulmonary hypertension (PH) development. Related to this, prior research has demonstrated the relevance of immune checkpoint protein programmed death ligand 1 (PD-L1) expression by immunoregulatory myeloid cells in PH. However, there remains a need to elucidate the cell-specific role of STING expression, and the STING/PD-L1 signaling axis in PH, before readily available disease-modifying therapies can be applied for patients with the disease. Here, through generation of bone marrow chimeric mice, we show that STING^–/–^ mice receiving WT bone marrow were protected against PH secondary to chronic hypoxia. We further demonstrate a cellular dichotomous role for STING in PH development, with STING expression by smooth muscle cells contributing to PH and its activation on myeloid cells being pivotal in severe disease prevention. Finally, we provide evidence that a STING/PD-L1 axis modulates disease severity, suggesting the potential for future therapeutic applications. Overall, these data provide evidence of STING’s involvement in PH in a cell-specific manner, establishing the biologic plausibility of developing cell-targeted STING-related therapies for PH.

## Abstract 2

The manuscript investigated the cell-specific role of STING in pulmonary hypertension development, as well as the STING/PD-L1 signaling axis in disease progression.

## Graphical abstract

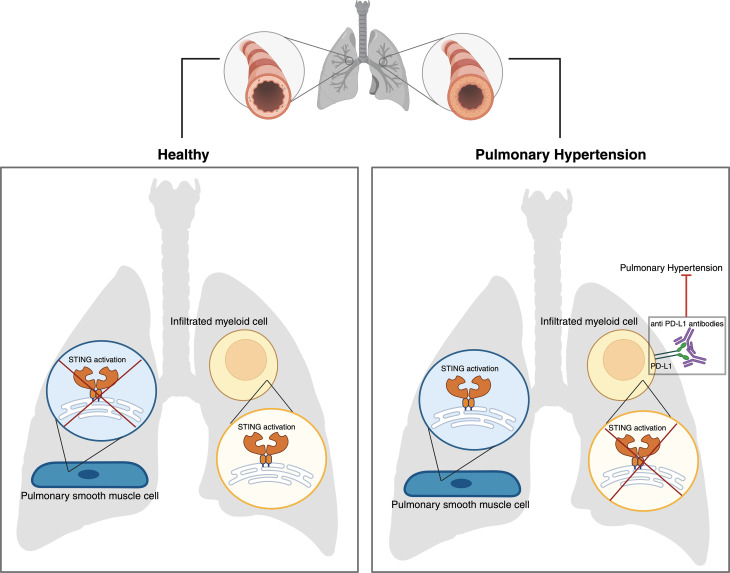

## Introduction

Pulmonary arterial hypertension (PAH) is a complex and progressive disease, with mortality acknowledged to be stagnant over the last 20 years (1). The extremely high mortality rate of 62% at 5 years after diagnosis (2) is associated with a notable reduction in quality of life related to major complications due to right heart failure and related comorbidities (3). WHO Group 3 pulmonary hypertension (PH) secondary to chronic lung disease and/or hypoxia also drastically worsens prognosis for patients with chronic lung disease, with escalating morbidity and mortality (4). The latter form of PH has thus become a disproportionate burden on the health care system, due to the increasing prevalence and complexity of management (5). With limited and often inadequate treatment for affected patients, more research is needed for further insight into general PH disease pathogenesis and potential therapies.

PH to date has been primarily studied in the context of homeostatic loss of function in resident lung cells, primarily pulmonary arterial endothelial cells (PAECs) (6, 7) and pulmonary vascular smooth muscle cells (PVSMCs) (8). Concurrently, PH is also characterized by chronic inflammation, with an influx of various innate and adaptive immune cells (9, 10). There is a pressing need to further study these cell-specific mechanism in PH in order to elicit better understanding of disease pathogenesis and development of effective disease-modifying therapy. To that end, our group has recently demonstrated the role of stimulator of interferon genes (STING), a cytosolic DNA sensor, in PH development (11). Interestingly, STING expression and activation in both pulmonary resident and pulmonary infiltrated immune cells have been shown to contribute to various disease pathogeneses that share the chronic inflammatory milieu described in relation to PH (12–15). We therefore hypothesized that STING contributes to PH in a cell-specific manner.

At baseline, pulmonary resident cells are critical for maintaining homeostasis of the lung microenvironment (16). STING expression on tissue-resident cells, such as PVSMCs and PAECs, have been shown to contribute to both immune activation and tissue remodeling in other vascular diseases (17, 18). This may shed light on the pathogenic role of pulmonary infiltrated inflammatory cells in PH development and progression (19). In particular, myeloid-derived suppressor cells (MDSCs) have been found to traffic to the lung from the bone marrow during PH development in mice (19), correlating well with clinical data showing an increased level of circulating MDSCs in patients with PH (20). Mechanistically, expression of immune checkpoint proteins on these cell populations has been found to correlate with disease severity, although regulation of expression has not been described (21). Recent studies also reported the crucial role of MDSC expression of STING in diseases characterized by chronic inflammation, such as cancer (22), as well as a novel role of STING in regulating immune checkpoint proteins (23). There is thus a need to investigate cell-specific role of STING in disease context in order to refine knowledge regarding the role of immune checkpoints in PH. This is especially important regarding the known pulmonary toxicities of the drug class, with immune checkpoint therapies (such as anti–PD-L1 antibody) posing a high risk of associated pneumonitis (24).

Herein, we report hypothesis-generating variations in myeloid STING expression in peripheral blood samples from PAH patients. We describe how STING regulates PH development and progression in a cell-specific manner. Specifically, we demonstrate that smooth muscle expression of STING contributes to disease pathogenesis, and that STING expression on myeloid cells is necessary to prevent development of severe PH. Last, we provide evidence of STING-dependent PD-L1 expression on myeloid cells as a marker for both disease severity, as well as a therapeutic target for patients.

## Results

### Patients with PAH display increased STING expression on pulmonary myeloid cells.

**Figure 1:**
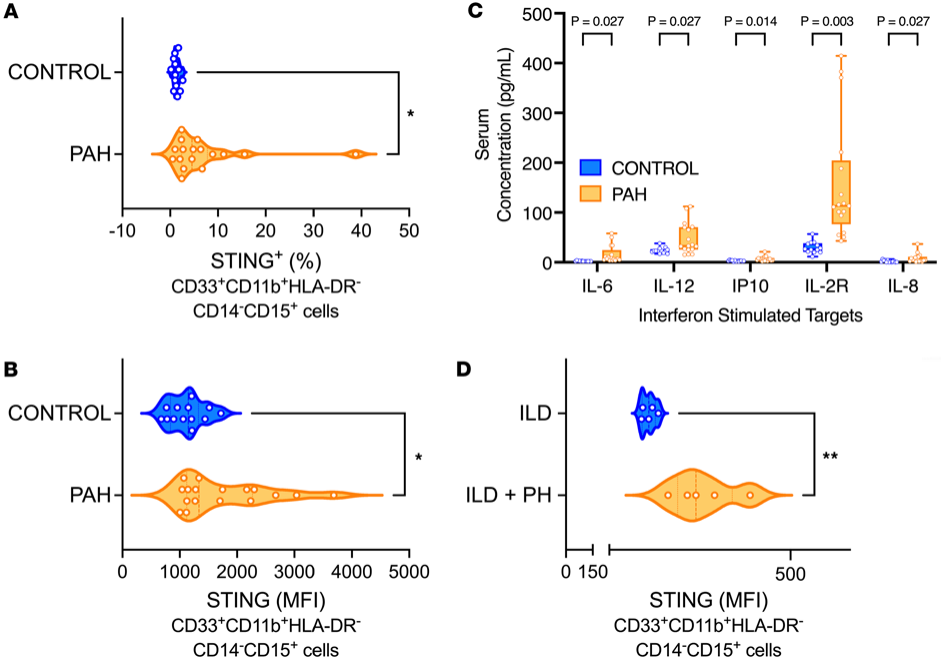
Patients with PAH display increased STING expression on pulmonary infiltrated myeloid cells. (**A**) Percentage and (**B**) MFI quantification of STING expression on circulating CD33^+^CD11b^+^HLA–DR^–^ cells of control and patients with PAH. (**C**) Serum concentration of interferon-stimulated interleukins from healthy individuals and patients with PAH. (**D**) MFI quantification of STING expression on circulating CD33^+^CD11b^+^HLA–DR^–^CD14^–^CD15^+^ cells of ILD patients with and without PH. Each dot represents an individual donor (*n* = 13–17/group, except for ILD cohorts, where *n* = 5/group). Violin plots show mean, mode, and interquartile range of the data set. Significance level was calculated with ANOVA followed by unpaired 2-tailed Welch’s *t* test corrected for multiple comparisons by use of Dunnett’s test, except for comparison of inflammatory target serum concentrations, where 2-way ANOVA was used with Bonferroni’s method to correct for multiple comparisons. *P < 0.05, **P < 0.01. *P* values are shown in the graph in **C**.

To establish the clinical relevance of STING expression to PH in a cell-specific manner, we reanalyzed a previously published single-cell RNA-Seq (scRNA-Seq) dataset from lungs of patients with PAH (compared with control donors without disease; *n* = 3/group) (25) for differential STING expression across common pulmonary cell types. We found varied expression across hematopoietic lineage and non–hematopoietic lineage cell populations (Supplemental Figure 1, A and B; supplemental material available online with this article; https://doi.org/10.1172/jci.insight.184792DS1), with gene ontology (GO) analysis notably indicating enrichment of STING-associated inflammatory signaling pathways across multiple cell subpopulations (Supplemental Figure 1C). We then conducted a pilot study to investigate the relevance of cell-specific STING expression. Using PBMCs from healthy individuals and patients with PAH (*n* = 13–17/group), we analyzed STING expression by flow cytometry quantification in various subpopulations of circulating cells. Notably, there was an increase in STING expression in CD33^+^CD11b^+^HLA–DR^–^ cells, consistent with surface markers attributed to MDSCs (26) (Figure 1, A and B; gating strategy previously described) (11). In addition, altered STING expression in these cells was accompanied by elevated serum concentration of interferon-stimulated interleukins, including IL-6, IL-12, IP10, IL-2R, and IL-8 in patients with PAH (Figure 1C). Additionally, we explored corollary findings in a separate pilot population of PH patients: those with PH secondary to interstitial lung disease (ILD). We found an increase in STING expression in a relevant subpopulation of MDSCs characterized by expression markers CD33^+^CD11b^+^HLA–DR^–^CD14^–^CD15^+^, known as polymorphonuclear MDSCs (PMN-MDSCs; Figure 1D). Finally, in a purely subjective hypothesis-generating fashion, we looked for nonhematopoietic cell lineage–dependent (endothelial cell and myofibroblast) qualitative differences in STING expression in histologic samples from healthy controls donors and patients with PAH and idiopathic pulmonary fibrosis (IPF) with or without PH (Supplemental Figure 2, A and B; control donor and patient demographics in Supplemental Table 1). STING was expressed by both cell types in patient lung samples. From these data, we hypothesized the potential cell-specific relevance of STING in disease pathogenesis, specifically within hematopoietic lineage cells, as this population has previously been reported to be important in patients with PH (27).

### Divergent roles of STING expression on hematopoietic and nonhematopoietic cells in PH.

**Figure 2:**
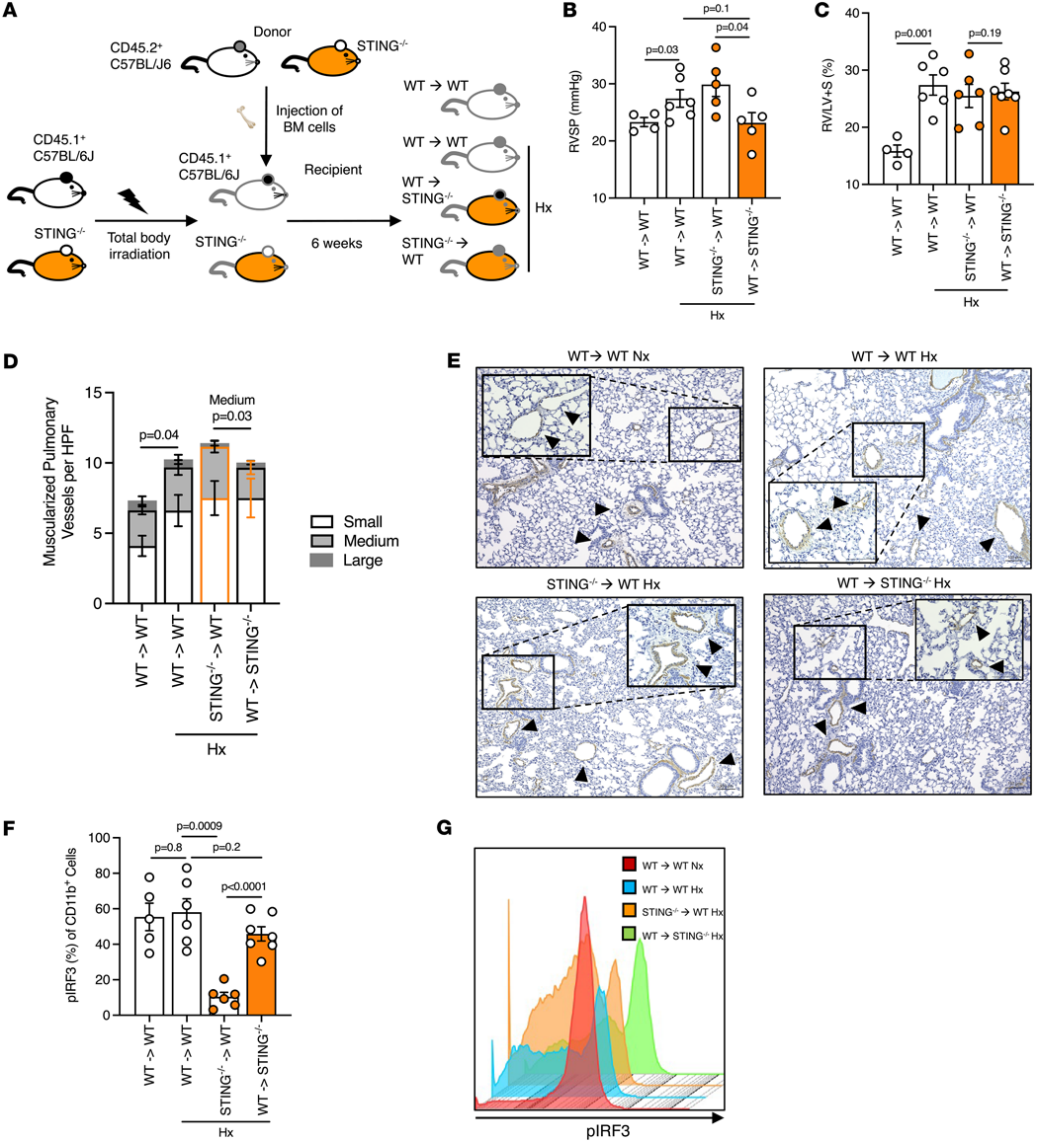
Divergent roles of STING expression on hematopoietic and nonhematopoietic cells in PH. (**A**) Schematic of the generation of control (WT) and STING-KO (STING^–/–^) chimeric mice in the indicated experimental groups. (**B**) RVSP measurement in WT and STING^–/–^ chimeric mice exposed to normoxia (Nx) or chronic hypoxia (Hx). (**C**) Fulton index (right ventricular mass over left ventricular plus septum mass [RV/LV+S]) of chimeric mice in the indicated experimental groups. (**D**) Quantification of small, medium, and large muscularized pulmonary vessels of experimental chimeric mice, assessing through α-SMA IHC staining. (**E**) Representative images of α-SMA (brown, arrowheads) IHC staining, with insets, of formalin-fixed lung sections from chimeric mice from the indicated experimental groups. Scale bars: 100 μm at 10× and 20× magnification. (**F**) Percentage and (**G**) flow representation of pIRF3 expression on CD11b^+^ cells from the different experimental groups. Each dot represents an individual mouse (*n* = 4–6/group). Data in bar graphs are presented as mean ± SEM. Significance levels were calculated with ANOVA followed by unpaired 2-tailed Welch’s *t* test corrected for multiple comparisons by use of Dunnett’s test. *P* values are shown in the graphs. *P* values in **F** were calculated from total vessels (small + medium + large) of mice from the experimental groups, unless specified otherwise.

To explore a potential hematopoietic cell–dependent role for STING in PH development, we generated bone marrow chimeric mice with either WT C57BL/6J or STING^–/–^ hematopoietic cells, on a WT C57BL/6J or STING^–/–^ nonhematopoietic background (Figure 2A). Donor mice were either CD45.2^+^ C57BL/6 or STING^–/–^, while recipient mice were either standard CD45.1^+^ C57BL/6J (WT) or STING^–/–^ mice that underwent whole body radiation. Chimerism efficiency was analyzed 6 weeks after bone marrow injection (Supplemental Table 2). Of note, the control group combination of STING^–/–^ donor and STING^–/–^ recipient (STING^–/–^→STING^–/–^) was uniformly lethal in mice prior to study inclusion, consistent with prior reports of model susceptibility to radiation therapy (28–30). Upon PH induction via chronic hypoxia exposure, a decrease in right ventricular systolic pressure (RVSP) was observed in STING^–/–^ mice receiving WT bone marrow cells (i.e., WT hematopoietic cells in a STING^–/–^ recipient), compared with WT mice receiving STING^–/–^ bone marrow cells (Figure 2B), although without noteworthy observed changes in RV remodeling (Figure 2C). An important caveat is that we did not assess the common model for PH induction Sugen/Hypoxia in reported studies, as our group has previously demonstrated that this model’s associated phenotype is itself dependent upon STING signaling (31). In alignment with our prior report, assessment of pulmonary vessel remodeling by α smooth muscle actin (α-SMA) staining revealed a decrease in medium-sized muscularized pulmonary vessels in STING^–/–^ mice receiving WT bone marrow cells compared with WT mice receiving STING^–/–^ hematopoietic cells (Figure 2, D and E). Given the observation of worsened RVSP in mice lacking hematopoietic STING activity, and previously published data showing that global STING-KO mice are protected against chronic hypoxia-induced elevated RVSP, we expected to see an attendant increase in STING activity in infiltrated inflammatory cells of mice with preserved STING expression compared with bone marrow–KO recipients, as assessed via an increase in phosphorylation of the important downstream transcription factor IRF3. However, we instead detected a statistically significant reduction of pIRF3 in WT mice receiving STING^–/–^ bone marrow cells compared with other groups (Figure 2, F and G), although no change in the number of infiltrated inflammatory cells was observed (Supplemental Figure 3, B–D; gating strategy shown in Supplemental Figure 3A). From this, we hypothesized that conditional knockout of STING in hematopoietic cells could potentially worsen PH. As an interim step based on a summary of our chimera data, we generated two hypotheses: first, that STING contributes to PH in a non–hematopoietic lineage–dependent manner; and second, that STING expression and activation in hematopoietic cells protect against PH.

Given that our group and others have previously demonstrated the relevance of immunoregulatory cells (MDSCs) in pulmonary vascular disease (19, 21, 32), and in combination with the observation that STING^–/–^ mice are protected against PH with an attendant decrease in the number of pulmonary infiltrated myeloid cells (11), we next sought to investigate deeper a cell-specific role of STING in PH development. We therefore performed scRNA-Seq, comparing whole lung cells of 8-week-old WT and STING^–/–^ mice exposed to either normoxia or chronic hypoxia (*n* = 2/group). Notably, given the known pathogenic roles of both endothelial (6) and mesenchymal cells (8) in PH, our scRNA-Seq data showed little change in endothelial cell populations of STING^–/–^ mice compared with WT mice undergoing chronic hypoxia exposure (Supplemental Figure 4C). However, consistent with the latter mentioned role of PVSMCs in PH, we found relevant expected differences in transcriptomes of smooth muscle cells between genotypes, as well as various other subpopulations of stromal cells (Supplemental Figure 4A). Additionally, in agreement with our bone marrow chimera data — which hinted at a potential protective role for STING expression in myeloid-derived cells — we detected an increased number of infiltrated myeloid cells, specifically nonclassical monocytes, and neutrophils, mirroring decreases in the inflammatory milieu of chronic hypoxia–exposed STING^–/–^ mice (Supplemental Figure 4B). Therefore, based on these data, we identified smooth muscle cells (nonhematopoietic) and myeloid cells (hematopoietic) as top candidates for the next steps in exploring the cell-specific role of STING in PH development.

### Smooth muscle cell–specific, but not endothelial cell–specific, deletion of STING provides protection against PH development secondary to chronic hypoxia.

**Figure 3:**
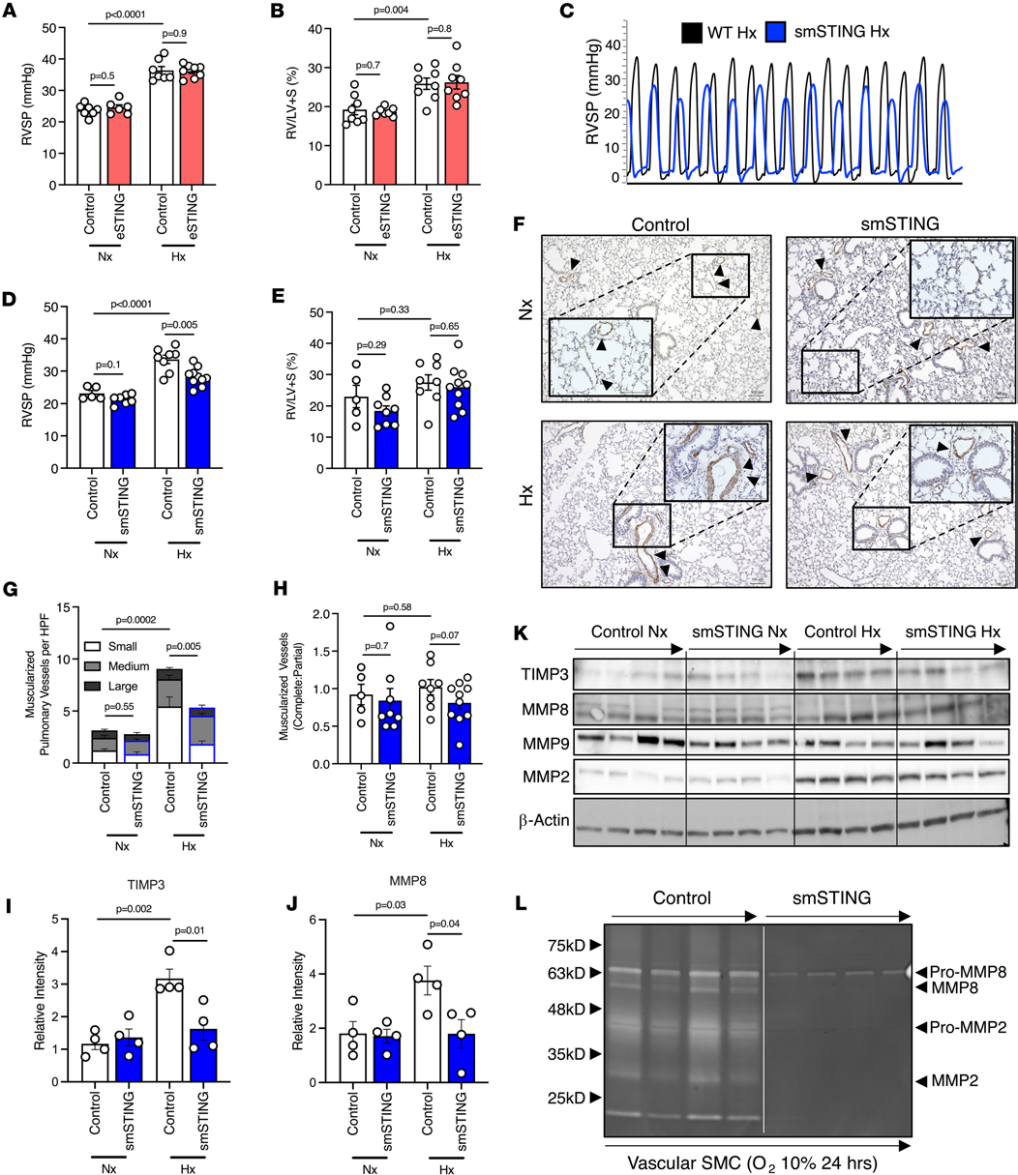
Smooth muscle, not endothelial, cell–specific deletion of STING provides protection against PH development secondary to chronic hypoxia. (**A**) RVSP measurements of littermate control (WT) and endothelial cell–specific STING-deficient (eSTING) mice exposed to normoxia or chronic hypoxia. (**B**) Fulton index of WT and eSTING mice from the indicated experimental groups. (**C**) RVSP curve representation and (**D**) RVSP measurement of littermate control (WT) and smooth muscle–specific STING-deficient (smSTING-deficient) mice exposed to normoxia or chronic hypoxia. (**E**) Fulton index of WT and smSTING mice in the indicated experimental groups. (**F**) Representative images of α-SMA (brown, arrowheads) IHC staining, with insets, of formalin-fixed lung sections from mice across indicated experimental groups. Scale bars: 100 μm. Original magnification ×10. (**G** and **H**) Quantification of (**G**) small, medium, and large and (**H**) completely or partially muscularized pulmonary vessels of WT and smSTING mice across experimental groups, as assessed through α-SMA IHC staining. (**I** and **J**) Quantification of Western blot of (**I**) TIMP3 and (**J**) MMP8 of smSTING and littermate control mice from different experimental groups. Each dot represents an individual mouse (*n* = 4–10/group). (**K**) Corresponding Western blots from mice in the indicated experimental groups (*n* = 4/group). (**L**) Gelatin zymography detection of pro-MMP2, MMP2, pro-MMP9, and MMP9 in lysates from isolated vascular smooth muscle cells (Vascular SMC) exposed to hypoxic conditions (10% ambient O_2_) for 24 hours (*n* = 4/group). Data are presented as mean ± SEM. Significance levels were calculated with ANOVA followed by unpaired 2-tailed Welch’s *t* test corrected for multiple comparisons by use of Dunnett’s test. *P* values are shown in the graphs. *P* values in **G** were calculated from total vessels (small + medium + large) of mice from the experimental groups, unless specified otherwise.

To test our working hypotheses, we first generated 2 primary transgenic mice with cell-specific deletion of STING to evaluate the nonhematopoietic role of STING in PH development. VE-cadherin–Cre (endothelial cell specific) and α-SMA–Cre (smooth muscle cell specific) mice were crossed to STING^fl/fl^ mice to produce mice with either endothelial cell–specific or smooth muscle cell–specific deletion of STING (referred to hereafter as “eSTING” and “smSTING,” respectively). Verification of endothelial cell deletion of STING is provided in Supplemental Figure 5. Consistent with our scRNA-Seq data regarding an absence of endothelial cell transcriptomic changes, there was no difference in RVSP or Fulton index between littermate control (WT) and eSTING mice subjected to chronic hypoxia (Figure 3, A and B). Given the lack of physiologic changes, there was, unsurprisingly, no change in the lung immune cell profile (i.e., CD11b^+^, CD11b^+^Ly6C^hi^Ly6G^–^, and CD11b^+^Ly6C^lo^Ly6G^+^) of eSTING mice (Supplemental Figure 6, A–C).

Given these data, we next characterized our smooth muscle cell–specific deletion model phenotype, subjecting 8- to 10-week-old smSTING mice to chronic hypoxia. Notably, the smSTING mice were protected against RVSP elevation upon chronic hypoxia exposure (Figure 3C). Although there was no substantial change in right ventricular hypertrophy as assessed with Fulton index between WT and smSTING mice exposed to chronic hypoxia (Figure 3, D and E), hypoxic smSTING mice demonstrated a lower level of muscularized pulmonary vessels, specifically small vessels, and a trend toward less complete muscularization compared with hypoxic WT mice (Figure 3, F–H). In whole lungs of the hypoxic smSTING mice, there was an association with a decrease in expression of the extracellular matrix (ECM) remodeling proteins TIMP3 and MMP8 (Figure 3, I–K), but not MMP9 and MMP2 (Supplemental Figure 6, D and E), compared with control. Gel zymography further demonstrated that among isolated vascular smooth muscle cells exposed to hypoxic conditions, cells from smSTING mice had markedly less MMP functional activity compared with controls (Figure 3L). STING deletion was confirmed in isolated smooth muscle cells from the smSTING-transgenic model (Supplemental Figure 6F). Finally, GO enrichment analysis revealed downregulation of genes involved in ECM remodeling in smooth muscle cells of hypoxic smSTING compared with hypoxic control mice (Supplemental Figure 6G). Thus, in aggregate, we conclude from these data a pathogenic role for smooth muscle STING in PH development, consistent with the previously described involvement of STING in ECM regulatory pathways contributing to cardiovascular disease (12).

### STING expression by myeloid cells is necessary to prevent severe PH.

**Figure 4:**
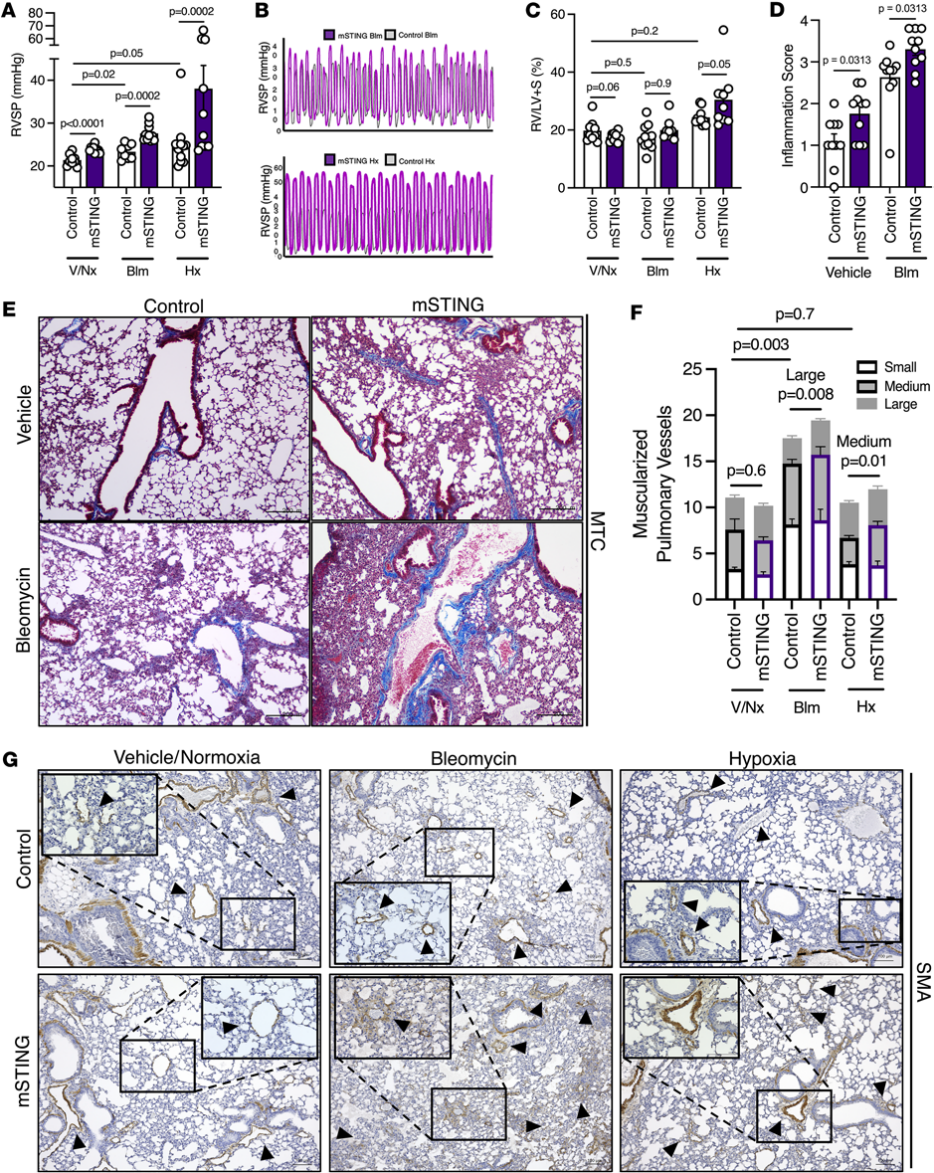
STING expression on myeloid cells is necessary to prevent severe PH. (**A** and **B**) RVSP measurements and curve representation of littermate control (WT) and myeloid-specific STING-deficient (mSTING) mice subjected to normoxia (Nx), bleomycin (Blm), or chronic hypoxia (Hx). (**C**) Fulton index of WT and mSTING mice from the indicated groups. (**D**) Inflammation/fibrosis quantification assessed through MTC-stained lung sections from WT and mSTING mice with or without bleomycin treatment. (**E**) Representative images of MTC-stained, formalin-fixed lung sections from WT and mSTING mice in the indicated groups at 10× magnification. Scale bars: 200 μm. (**F**) Quantification of small, medium, and large muscularized pulmonary vessels from WT and mSTING mice across the experimental groups, as assessed through α-SMA IHC staining. (**G**) Representative images of α-SMA (brown, arrowheads) IHC staining, with inset, of formalin-fixed lung sections from mice across the indicated experimental groups. Scale bars: 100 μm at 10× magnification. Each dot represents an individual mouse (*n* = 5–10/group). Data are presented as mean ± SEM. Significance levels were calculated with ANOVA followed by unpaired 2-tailed Welch’s *t* test corrected for multiple comparisons by use of Dunnett’s test. *P* values are shown in the graphs. *P* values in **F** were calculated from total vessels (small + medium + large) of mice from the experimental groups, unless specified otherwise.

Having identified a pathogenic role for smooth muscle STING in PH, we next sought to understand the role of myeloid cell STING expression in PH development. To test our working hypothesis that STING expression by myeloid cells prevents development of severe PH, we crossed LysM-Cre mice with STING^fl/fl^ mice (referred to as “mSTING”) to generate progeny with myeloid cell–specific deletion of STING. Verification of cell-specific deletion of STING is provided in Supplemental Figure 7. Given prior work with this relatively cell-specific model in both bleomycin-induced and chronic hypoxia–induced PH (19, 21, 27, 33) demonstrating that the MDSC suppressive phenotype is strongest when the i.p. bleomycin model is used, 8- to 10-week-old mSTING mice were subjected to PH induction using both of these physiologically complementary models. Consistent with our prediction that hematopoietic-lineage STING expression may have a protective role against PH, mSTING mice developed severe PH under both bleomycin and chronic hypoxia exposure (Figure 4, A and B). Right ventricular remodeling, assessed by Fulton index, was increased in mSTING mice exposed to chronic hypoxia (Figure 4C). Of note, at baseline mSTING mice displayed higher RVSP compared with control mice, signifying a potentially important role for myeloid STING expression in maintenance of pulmonary vascular homeostasis at baseline (Figure 4A). As expected, based on the elevation in RVSP, an increase in inflammation/fibrosis was observed in the myeloid cell–specific mice treated with bleomycin compared with littermate controls (Figure 4, D and E). Also, consistent with the physiologic data, we saw an appreciably higher degree of tissue scarring and collagen deposition in mSTING mice after bleomycin exposure and at baseline (Figure 4, D and E). Given this discrepancy, we cannot ultimately rule out a contribution from parenchymal fibrosis in the development of pulmonary vascular remodeling, with associated vascular rarefaction; however, an argument against this conclusion is that assessment of muscularized pulmonary vessels demonstrated an actual increase in medium- and large-sized vessels in mSTING mice subjected to bleomycin and chronic hypoxia, respectively (Figure 4, F and G).

**Figure 5:**
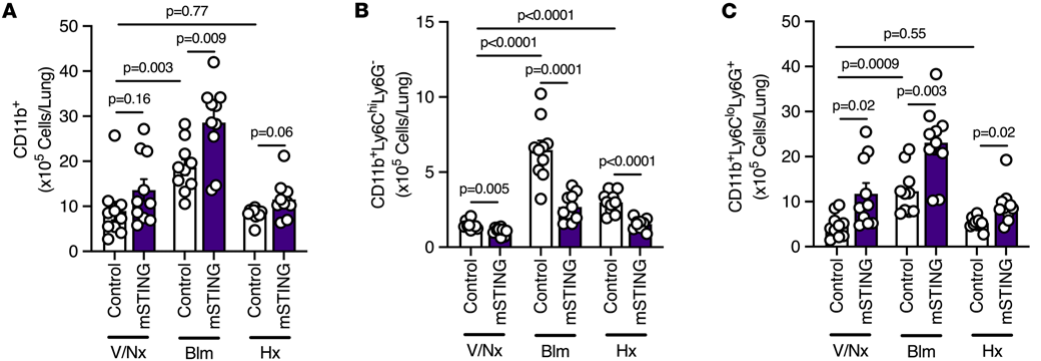
mSTING mice subjected to bleomycin or chronic hypoxia display an increase in pulmonary infiltrated proinflammatory cells. (**A**–**C**) Flow cytometric quantification of pulmonary infiltrated (**A**) CD11b^+^, (**B**) CD11b^+^Ly6C^hi^Ly6G^–^, and (**C**) CD11b^+^Ly6C^lo^Ly6G^+^ cells of littermate control and smSTING mice from different experimental groups. Each dot represents an individual mouse (*n* = 5–10/group). Data are presented as mean ± SEM. Significance levels were calculated with ANOVA followed by unpaired 2-tailed Welch’s *t* test corrected for multiple comparisons by use of Dunnett’s test. *P* values are shown in the graphs.

Upon flow cytometric analysis of lungs, we noted an increase in CD11b^+^ cells in mSTING mice subjected to either bleomycin or chronic hypoxia compared with littermate controls (Figure 5A and Supplemental Figure 8A). Interestingly, there was a decrease in number of protective CD11b^+^Ly6C^hi^Ly6G^–^ cells in mSTING mice in severe PH, consistent with our previously published findings across a variety of preclinical models (21, 33) and peripheral blood samples from patients with PAH (34) (Figure 5B and Supplemental Figure 8B). As expected, based on worsened PH, however, both bleomycin-treated and hypoxic mSTING mice displayed an increase in historically deleterious CD11b^+^Ly6C^lo^Ly6G^+^ cells (Figure 5C and Supplemental Figure 8B). Of note, there was a small yet statistically significant decrease in phagocytosis capability by differentiated macrophages from untreated mSTING compared with control mice (Supplemental Figure 8C). Thus, we cannot definitively rule out an overall increase in lung inflammation related to depressed phagocytosis. In total, however, these data demonstrate that myeloid STING is important in the prevention of severe PH via regulation of inflammatory cells within the lung.

### Severity of PH in mSTING mice is PD-L1 dependent.

**Figure 6:**
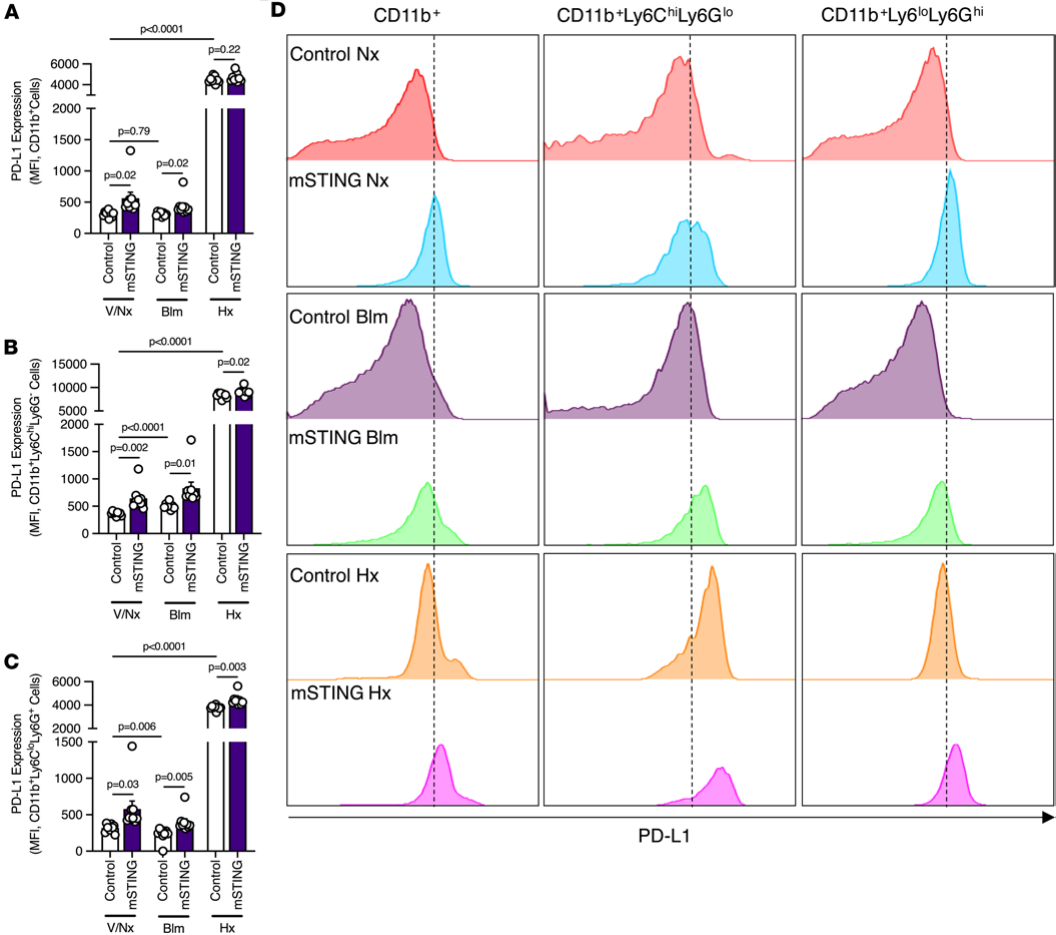
Severity of PH in mSTING mice is PD-L1 dependent. (**A**–**C**) Flow cytometric quantification of PD-L1 expression in pulmonary infiltrated (**A**) CD11b^+^, (**B**) CD11b^+^Ly6C^hi^Ly6G^–^, and (**C**) CD11b^+^Ly6C^lo^Ly6G^+^ cells of littermate control and mSTING mice from the different experimental groups. (**D**) Flow chart representation of PD-L1 expression in the indicated cell populations across experimental groups. Each dot represents an individual mouse (*n* = 5–10/group). Data are presented as mean ± SEM. Significance levels were calculated with ANOVA followed by unpaired 2-tailed Welch’s *t* test corrected for multiple comparisons by use of Dunnett’s test. *P* values are shown in the graphs.

Given that myeloid cell–specific deletion of STING caused severe PH in mice, we asked whether there was a separate STING-associated mechanism specific to myeloid cells, in particular MDSCs, that could explain the worsening in pulmonary vascular remodeling. We have previously demonstrated that MDSCs in patients with PH display a markedly increased level of PD-L1 expression, correlating with disease severity, a result that we later were able to phenocopy in mice, with higher PD-L1 expression on MDSCs reflecting an increase in suppressive capability and worsened PH (21). Related to this, STING activation in MDSCs has been shown to activate antitumor immunity (35). Based on these previous findings, we set out to examine PD-L1 expression on CD11b^+^Ly6C^hi^Ly6G^lo^ and CD11b^+^Ly6C^lo^Ly6G^+^ cells in mSTING mice. We hypothesized that these mice with more severe PH would exhibit higher PD-L1 expression on myeloid cell subpopulations of interest. Supporting this hypothesis, these mice demonstrated a statistically significant increase in PD-L1 expression on CD11b^+^, CD11b^+^Ly6C^hi^Ly6G^–^, and CD11b^+^Ly6C^lo^Ly6G^+^ cells after induction of PH with bleomycin (Figure 6, A–D). Similar changes were observed in hypoxic mSTING mice, specifically in the CD11b^+^Ly6C^hi^Ly6G^–^ and CD11b^+^Ly6C^lo^Ly6G^+^ cell populations (Figure 6, A–D), although conclusions regarding MDSC subtype–specific effects on PH severity cannot be drawn from current data. Notably, however, there was an increase in PD-L1 expression on lung myeloid cells at baseline in mSTING mice, consistent with the elevation in RVSP displayed in these mice (Figure 6, A–D). Therefore, in summary of the data to this point, we concluded that PD-L1 expression on myeloid cells was STING dependent in PH, correlative with disease severity. We therefore next sought to demonstrate whether there was a protective role for use of PD-L1 inhibitors in mSTING mice.

### Anti–PD-L1 antibody abrogates severe PH in mSTING mice.

**Figure 7:**
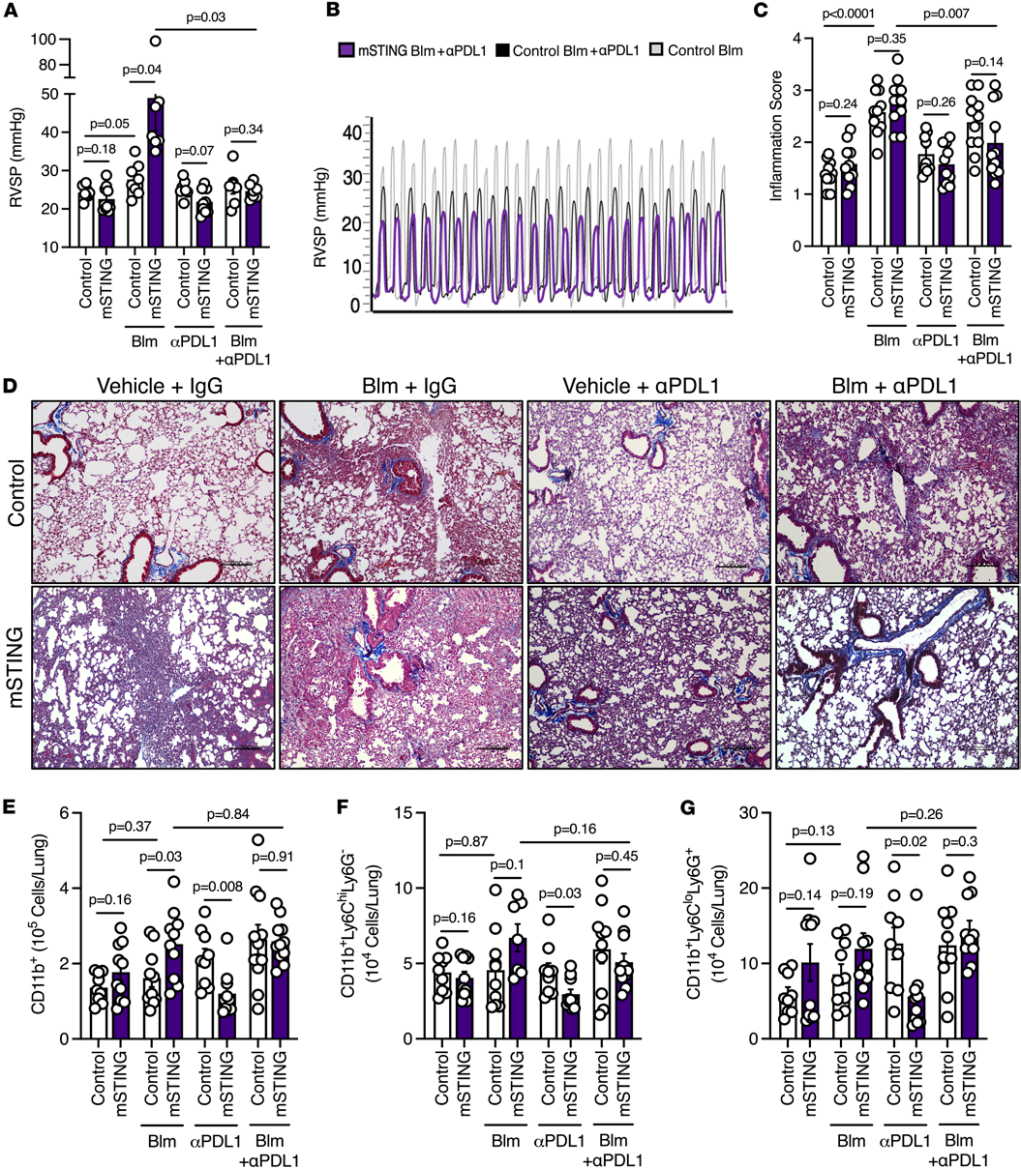
Anti–PD-L1 antibody abrogates severe PH in mSTING mice. RVSP (**A**) measurement and (**B**) curve representation of littermate control (WT) and mSTING mice subjected to vehicle or bleomycin and treated with IgG or anti–PD-L1 (αPD-L1) as indicated. (**C** and **D**) Quantification of inflammation/fibrosis degree and representative images of MTC-stained, formalin-fixed lung sections from WT and mSTING mice in the indicated experimental groups. (**E**–**G**) Flow cytometric quantification of pulmonary infiltrated (**E**) CD11b^+^, (**F**) CD11b^+^Ly6C^hi^Ly6G^–^, and (**G**) CD11b^+^Ly6C^lo^Ly6G^+^ cells from WT and mSTING mice across different experimental groups. Each dot represents an individual mouse (*n* = 5–13/group). Data are presented as mean ± SEM. Significance levels were calculated with ANOVA followed by unpaired 2-tailed Welch’s *t* test corrected for multiple comparisons by use of Dunnett’s test. *P* values are shown in the graphs.

In a preventive and proof-of-concept manner, we pretreated mice with a PD-L1–specific inhibitory antibody and exposed them to bleomycin. The chronic hypoxia model was not utilized to avoid an additional prolonged period of normoxia (due to injection schedule) in exposed mice. Building upon our prior findings (21), treatment with anti–PD-L1 in 8- to 10-week-old mSTING mice with bleomycin-induced PH attenuated the severe disease phenotype (Figure 7, A and B). Despite the clinically relevant concern for immune checkpoint inhibitor–induced (ICI-induced) pneumonitis, bleomycin-exposed mSTING mice with anti–PD-L1 therapy showed a noteworthy reduction in inflammation/fibrosis compared with WT mice (Figure 7, C and D), with an attendant decrease in muscularized large pulmonary vessels (Supplemental Figure 9, A–C). Reflecting these physiological data was the observed normalization in the quantity of inflammatory cells (i.e., CD11b^+^, CD11b^+^Ly6C^hi^Ly6G^–^, and CD11b^+^Ly6C^lo^Ly6G^+^ cells) in bleomycin-treated mSTING mice receiving anti–PD-L1 therapy (Figure 7, E–G). Thus, mice with myeloid cell–specific STING deficiency developed severe PH, a phenomenon that could be prevented by anti–PD-L1 therapy.

In summary, these data demonstrate evidence for a dichotomous role for STING in lung-resident (i.e., smooth muscle) and infiltrated inflammatory (i.e., myeloid) cells in PH development. In particular, PD-L1 expression correlated with disease severity in a cell-specific manner, with a STING/PD-L1 signaling axis showcasing the potential for cell-specific therapeutic intervention. Given the large amount of safe anti–PD-L1 therapies available on the market, future studies should assess the utility of this therapy in various groups of patients with PH.

## Discussion

PH is a devastating disease, with rising incidence and prevalence, and a 7.2-fold increase in 1-year standardized mortality ratio over the past 3 years (2). As there are no current disease-modifying therapies for patients with PH, there is an urgent need to identify specific disease mechanisms that can be therapeutically targeted. Here, we report a cell-specific role for the DNA-sensing protein STING in PH development. Using multiple PH models, we confirmed that STING activation regulates disease development, with changes in immune cell infiltration. Specifically, we provide evidence that smooth muscle cell expression of STING worsens PH, while STING expression by myeloid cells is critical to prevent development of severe PH. Furthermore, we show that alteration in myeloid STING induces PD-L1 expression on infiltrated myeloid cells with the potential to serve as a marker for disease severity as well as a therapeutic target. Overall, our results reinforce the notion that STING is an important regulator of PH development and progression, and thus have the potential to offer urgently needed cell-specific disease-modifying targeted pathways.

Our group has recently reported on the contribution of STING to PH (11), with global KO of STING providing protection against development of PH in several disease models. As STING is expressed in multiple cell types, and its function is altered depending on the tissue of interest, it is unsurprising based on our findings that STING plays a cell-specific role in PH. Given the literature to date on endothelial STING expression, however, it was surprising that the endothelial STING deletion model did not indicate a role for the protein in disease onset and progression. It is not entirely unexpected, however, that smooth muscle cell STING expression is important, as recent research has provided insight into a potential mechanism for STING promotion of vascular smooth muscle cell proliferation (36–38), a hallmark of PH progression. Related to this, we found that hypoxia-exposed smSTING mice protected against elevated RVSP displayed a decrease in expression of proteins crucial for ECM remodeling contributing to PH, including MMP8 (39) and TIMP3 (40). Previously, STING has been described as promoting ECM remodeling via regulation of MMP and TIMP expression, though in different vascular and inflammatory diseases (12, 41), not in PH. In addition to their roles in maintaining the structural balance of the ECM, MMPs and TIMPs have been shown to be involved in a multitude of inflammatory responses (42, 43), including immune cell influx into tissue (44). It is interesting to speculate then that STING expression on smooth muscle cells is crucial in ECM remodeling, as well as in recruitment and activation of inflammatory cells (i.e., myeloid cells), regulating pulmonary vascular remodeling as well as more general pulmonary fibrotic disease (45, 46). However, the exact mechanism of STING of ECM-related protein expression and activity requires further investigation.

While the pathogenic role of smooth muscle STING is interesting and consistent with described literature on the subject, the protective effect of myeloid STING in severe PH was surprising. Studies of STING in similar chronic inflammatory diseases with cellular features similar to those of PH, such as cancer, describe myeloid cell STING as pathogenic (47). For example, activation of STING in cancer leads to recruitment of MDSCs to the site of chronic “injury” (i.e., tumor) with described immunosuppressive activity (22). It is interesting to speculate that STING-mediated cell-cell interactions (mesenchymal and myeloid cells, specifically) may account for some of the disease-specific differences in presentation. The study of such cell-cell interactions in PH, either through paracrine signaling or as a field effect, is a subject of ongoing investigation in the field (48–50). Viral DNA-sensing pathways stand as a key mediator in research on these responses. Finally, such complexities in compensatory STING expression may also explain why in our reported data from patients with PAH, a noted increase in STING expression is seen (27). Of course, the finding could also represent an entirely unrelated epiphenomenal response in idiopathic Group 1 PAH, in contrast to the Group 3 PH models used in this study, an area of emerging research in the field (51, 52).

In contrast to established studies of STING agonist synergizing with checkpoint inhibitors in cancer immunotherapy (53, 54), blocking PD-L1 rescued mSTING mice from severe PH in the present studies. This represents a complex regulatory network in a different disease context that will require further research]. For example, there is a need to carefully distinguish the role of nuclear PD-L1 on STING activation, given a prior group’s demonstration that change in protein function was related to distinct cellular compartments (23). Such discernment may be relevant to interpretation of our findings, as we focused exclusively on PD-L1 surface expression and targeting. Overall, the exact mechanism of PD-L1–dependent STING regulation in myeloid cells is an ongoing and exciting area of research, exploring the role of STING in autophagic regulation of immune checkpoint expression and altering immune cell functions (55, 56).

Importantly, our study has several key limitations. Engraftment efficiency in our chimera experiment was directly influenced by the protein target of interest, STING, particularly influenced by the response to external radiation (22, 57, 58). Another clinically relevant weakness is that available therapeutic PD-L1 inhibitors contribute to development of severe pneumonitis in a meaningful number of patients (59). Though our studies demonstrated that bleomycin-induced lung injury in mSTING mice actually improved after PD-L1 antibody treatment, this will be an ongoing point of safety monitoring in any future clinical trial designs targeted for patients with Group 3 PH, especially those with IPF. It is worth noting, however, that patients with IPF and PH have a life expectancy shorter than patients with most solid malignancies, and in risk-benefit analysis, the latter group is routinely treated with ICI therapies as well as complex chemotherapeutic regimens targeting cell death in the treatment of primary lung cancer. The mechanism by which immune-checkpoint inhibitors may rescue PH thus requires further investigation and intense post-market monitoring before translation of effective therapies can be routinely administered (24, 59).

Finally, considering prior reports on the importance of endothelial cell STING expression for vascular health, it is necessary to emphasize the need for follow-up studies regarding the lack of protection against PH development in endothelial cell–specific STING-depleted mice. To our knowledge, there are only a handful of reports that explicitly present data from an endothelial cell–specific model of STING deletion. One such rigorous publication (60) presented results detailing that endothelial cell–specific STING-depleted mice are protected against chemotherapeutic-mediated cardiac toxicity due to a decrease in canonical STING-associated interferon signaling. The group goes on to demonstrate that this protective mechanism occurs via lack of downstream TBK1 binding and transcription of interferon-stimulated genes, with subsequent heart damage due to immune cell infiltration and activation. These findings are consistent with a previous report on endothelial cell STING utilizing a diabetic retinopathy model, activation of TBK1-mediated inflammatory disease (61). Both studies emphasize the canonical role of cGAS/STING in type I interferon–mediated disease, in contrast to the noncanonical signaling pathways that our group has examined to date. Thus, phenotypic discrepancies highlight the need for further consideration of cell-specific classical and nonclassical STING signaling in studies of disease detection and treatment.

In conclusion, our study provides evidence for a dichotomous role for STING in lung-resident and infiltrated inflammatory cells in PH. In addition, PH severity was shown to correlate with PD-L1 expression on myeloid cells, through a STING-dependent mechanism. Thus, cell-specific targeting of STING/PD-L1 may be of therapeutic value.

## Methods

### Sex as a biological variable

For studies using human and animal samples, sex is specified as appropriate to the design of the given experiment. For preclinical studies, age and sex-matched mice were used throughout, and analysis by individual sex did not reveal dimorphism in phenotype. Thus, data for males and females sexes have been combined.

### Mice

STING^–/–^ (MPYS^–/–^) mice were purchased from The Jackson Laboratory (strain 025805), then crossed with in-house C75BL/6J mice for maintenance. Pairs of STING^fl/fl^ (strain 035692), VeCad-Cre (strain 006137), LysM-Cre (strain 004781), and SMA-Cre (strain 029925) mice were purchased from The Jackson Laboratory and maintained in house with a C57BL/6J background. Thus, all transgenic mice generated in this study were on a C57BL/6J background. Transgenic mice expressing Cre-recombinase under the control of the VE-cadherin promoter (VeCad-Cre) were crossed with STING mice flanked by 2 loxP sites (STING^fl/fl^) to generate Cre-mediated specific deletion of the STING gene in endothelial cells. Similarly, transgenic mice expressing Cre combinase under the control of the LysM promoter (LysM-Cre) or SMA promoter (SMA-Cre) were crossed with STING^fl/fl^ mice for generation of Cre-mediated specific deletion of the STING gene in myeloid cells and smooth muscle cells, respectively. Breeding was set up such that the STING construct was kept in a homozygous state, while VeCad-Cre, LysM-Cre and SMA-Cre were maintained in a heterozygous state, yielding Cre^+^ mice with respective cell deletion. Cre^–^ mice were used as littermate controls. Mice were bred and housed in specific pathogen–free conditions. Eight- to 10-week-old sex-matched mice were used for each experiment (4–5 mice/sex/group), after confirmation of appropriate genotype, with genotyping performed for all experiments.

### Mouse models of PH

PH was induced in mice with either bleomycin injection or chronic hypoxia exposure.

#### Bleomycin.

Mice received i.p. injections of bleomycin (MilliporeSigma 9041934) at 0.018 U/g twice a week for 4 weeks. Weights of animals were monitored throughout the injection period. A 20% loss of body weight resulted in temporary termination of bleomycin treatment. Injection was resumed when the animal regained at least 10% of lost weight. Euthanasia and data collection were performed 5 days after final injection, on day 33 of the bleomycin injection protocol.

#### Chronic hypoxia.

Mice undergoing chronic hypoxia exposure were placed in a normobaric ventilated chamber, in which the level of O_2_ is controlled through flow of N_2_ (ProO_2_ monitor/controller and chamber, Biospherix). O_2_ and CO_2_ concentration was monitored continuously, such that their concentrations remained at 10% and 0.1% respectively. Exposure to normal air was limited to water, food, and cage changes. All mice were sacrificed after 28 days (4 weeks) of exposure for analysis.

### Generation of bone marrow chimeric mice

Ten-week-old recipient CD45.1^+^ and STING^–/–^ male mice were irradiated with 2 doses at 5 Gy (11 minutes, 4-hour interval) and received bone marrow cells from 10-week-old male CD45.2^+^ or STING^–/–^ donor mice the next day through retro-orbital injection. Antibiotic (TMS, 100 mg/kg) was delivered in drinking water together with soft food for 2 weeks after bone marrow transplantation. Six weeks were allowed for bone marrow reconstitution before mice were subjected to 4 weeks of chronic hypoxia exposure. An experimental design scheme is provided in Figure 2A.

Chimerism was confirmed using flow cytometry staining for CD45.1 or CD45.2 in recipient mouse submandibular blood collected at 6 weeks after transplantation. Chimerism was calculated as: % cells expressing CD45.2/% cells expressing CD45.1.

### Treatment with anti–PD-L1 antibody

Eight- to 10-week-old LysM-Cre^+/–^STING^fl/fl^ and LysM-Cre^–/–^STING^fl/fl^ male mice were given i.p. injections of 500 mg anti–PD-L1 antibody (BioXCell BE0101) or isotype control (BioXCell BE0090) once a week for 4 weeks, concurrently with injection of bleomycin (0.018 U/g twice a week) or PBS according to experimental group.

### Primer sequences in mice

See Supplemental Table 3 for primer sequences in mice. SAVI mice genotyping was sent to Transnetyx for confirmation of appropriate genotype.

### Pulmonary hemodynamic assessment

Mice were put under deep anesthesia with i.p. injection of 25% avertin (2,2,2-tribromoethanol, Thermo Fisher Scientific AC421432500) in PBS at a 16 mg/kg dose. A 1.4-French pressure-volume microtip catheter (Millar Instruments, SPR-839) was inserted through a right internal jugular incision and threaded down into the right ventricle. The catheter was connected to a signal processor (PowerLab and ADInstruments), and RVSP (mmHg) was recorded digitally and displayed with Chart5. After stable measurements of a minimum of 5 minutes, animals were euthanized, with hearts and lungs removed for subsequent analysis. The right ventricle was separated from the heart after removal of the atria, and the weights of both right ventricle (RV) and left ventricle plus septum (LV+S) were obtained. Right ventricular hypertrophy was later calculated using the ratio RV/LV+S (%) (Fulton index).

### Flow cytometry and phagocytosis assay

The left lung was cut into pieces and digested for 1 hour with 3 mL RPMI 1640 with 10% FBS (Gibco A4766801), DNAse I (10 mg/mL, Roche 10104159001), and 5% Liberase (MilliporeSigma 05401127001) at approximately 200*g* and 37°C. Tissues were triturated with 3 mL syringes and 18G needles until complete dissociation was achieved. Cells were filtered through 70 mm cell strainer, washed 3 times with D-PBS–2% FBS and 1 M EDTA to remove debris. Remaining red blood cells were lysed with ammonium chloride lysis buffer (KD Medical 50-1019080). Single-cell suspensions obtained were stained with fluorochrome-conjugated surface antibodies for 30 minutes on ice, Fixable Viability Dye (eBioscience 65-0865-14) for 30 minutes, or overnight on ice; and fixed, permeabilized, and then incubated with intracellular markers for 30 minutes on ice. Data were acquired using a BD FACSymphony A3 Cytometer (BD Biosciences) with 5 lasers and analyzed with FlowJo version 10 software. A list of antibodies is provided in Supplemental Table 4. Phagocytosis was assessed using the Vybrant Phagocytosis Assay Kit (Thermo Fisher Scientific V6694), per the manufacturer’s instructions, with isolation and macrophage differentiation in vitro as previously published by our group (31).

### FACS antibodies

A comprehensive list of FACS antibodies used is presented in Supplemental Table 4.

### cDNA library construction and scRNA-Seq

Mouse whole lungs were perfused with 10 mL PBS to remove blood cells. Lung tissues then were cut into small pieces and incubated in 3 mL RPMI 1640 with 10% FBS (Gibco A4766801), DNAse I (10 mg/mL, Roche 10104159001) and 5% Liberase (MilliporeSigma 05401127001) for 1 hour at Approximately 200*g* and 37°C. Tissues were triturated with 3mL syringes and 18G needles until complete dissociation was achieved. Cells were filtered through 70 mm cell strainer, washed 3 times with D-PBS–2% FBS and 1 M EDTA to remove debris. Remaining red blood cells were lysed with ammonium chloride lysis buffer (KD Medical 50-1019080). Single cells were captured in 10X Genomics Chromium Single Cell 3′ Solution, and RNA-Seq libraries were prepared following the manufacturer’s protocol (10X Genomics). The libraries were subjected to high-throughput sequencing on an Illumina NovaSeq 6000 platform, targeting 6,000–8,000 cells per sample with a sequencing depth of at least 20 million reads of 150 bp paired-end reads.

### Process and quality control of the scRNA-Seq data

The raw sequencing reads were aligned with mouse genome mm10 provided on the CellRanger website by 10X Genomics. The mapped reads then were used for unique molecular identifier (UMI) counting, following the standard CellRanger pipeline for quality control as recommended by the manufacturer (10X Genomics). In short, cells with UMI counts lower than 500 or a feature count less than 200 were excluded. In addition, cells with greater than 30% of RNA content made up of the most common genes were excluded, as they accounted for empty droplets with free-floating RNA. Cells with greater than 30% of RNA content mapped to the mitochondrial genome were also eliminated, as they indicated poor quality. Last, cells with abnormally high counts were discarded. Subsequently, the filtered single cells were imported into the R package “Seurat” (version 4.0) for clustering of data and calculating differential gene expression, following the standard pipeline per the manufacturer’s instruction (10X Genomics). Markers for different populations of cells (stromal, endothelial, and myeloid) were obtained from previously described work (62–66).

### Mouse sample histological staining

The right lower lobe of mouse lungs, upon harvest, was fixed in formalin overnight. Fixed tissues were paraffin embedded, cut with a Leica RM2235 Microtome, and stained for Masson’s trichrome (MTC) and α-SMA to assess inflammation as well as to identify muscularized pulmonary vessels (21).

#### MTC staining and semiquantitative inflammation scoring.

Lung inflammation was evaluated on trichrome-stained lung sections using a 0–4 scale: score of 0, normal lung architecture; 1, increased thickness of some (<50%) of the interalveolar septa; 2, thickening of >50% of the interalveolar septa without formation of fibrotic foci; 3, thickening of the interalveolar septa with formation of isolated fibrotic foci; and 4, formation of multiple fibrotic foci with total or subtotal distortion of parenchymal architecture. Masked evaluation was performed on 10 randomized sequential, nonoverlapping fields (magnification 10×) of lung parenchyma from each specimen. The mean score for the 10 fields represented the score for each individual specimen.

#### a-SMA staining and muscularized vessel count.

Formalin-fixed lung sections were stained for rabbit polyclonal α-SMA (Abcam ab5694; diluted 1:750 in antibody diluent reagent solution [Life Technologies], not reused; blocking reagent, Background Sniper [Biocare]). Stained lung specimens were then randomized, and lung parenchyma from each specimen was assessed in a masked manner for pulmonary vessel counts in 10 sequential, nonoverlapping fields (magnification, 10×). Partially or completely muscularized pulmonary vessels were visualized in brown. Vessels were considered small if length was less than 50 μm; medium if 50–150 μm; and large if greater than 150 μm.

For immunofluorescence staining, sections were incubated with the primary antibodies CD31 (R&D Systems, AF3628, 1:100) or CD11b (Novus, NB600-1327, 1:100) and STING (Novus, NBP2-24683, 1:100) for 1 hour at room temperature. Sections were then incubated with corresponding secondary antibodies, prior to being mounted using Vectashield Vibrance Antifade DAPI (Vector Laboratories), with imaging as described below.

### Image processing and acquisition

Images of representative tissues from the histological staining were taken with a Keyence BZ-X microscope at 10×–20× magnification. Image processing was performed using BZ-X-Analyzer software (Keyence).

### Western blot and zymography

Right middle lobes were cut into pieces in 1 mL Pierce RIPA Buffer (Thermo Fisher Scientific 89901), 1X Halt Protease & Phosphatase Inhibitor Cocktail (Thermo Fisher Scientific 1861282), then homogenized using QIAGEN TissuelyzerLT. Protein concentration was determined using Pierce BCA Protein Assay Kits (Thermo Fisher Scientific 23225). 10% Criterion TGX Precast Gels (Bio-Rad 5671034) were used for protein separation (20 μg per sample) in Criterion Midi Cell (Bio-Rad 1656001) with 1× Tris/Glycine Buffer (Bio-Rad 1610771). Proteins were transfered to membrane blots using the Trans-Blot Turbo Transfer System (Bio-Rad 1704150) at standard setting. Membranes were blocked with EveryBlot Blocking Buffer (Bio-Rad 12010020) for 10 minutes at room temperature, then incubated with primary antibodies (1:1,000) against MMP8 (ProteinTech 17874-1), MMP9 (Cell Signaling Technology 24317AS), MMP2 (Cell Signaling Technology 87809S), TIMP3 (Cell Signaling Technology 5673S), STING (Cell Signaling Technology 13647), and β-Actin (Cell Signaling Technology 4967L) at 4°C overnight. Membranes were then washed in TBS 0.1% Tween 20 and incubated with secondary antibodies (1:10,000) for 1 hour at room temperature. Protein expression was detected with Clarity ECL Western Blotting Substrates (Bio-Rad 1705060) and visualized with the ChemiDoc Imaging System (Bio-Rad 12003153). Quantification of protein was performed in Image Lab software (Bio-Rad). All protein levels were normalized to the β-Actin protein level.

Primary vascular SMCs were isolated from smSTING and control mice as previously described (67). Cells were then grown to confluence in 6-well plates before being exposed to hypoxic conditions for 24 hours (10% O_2_ concentration; O_2_ Control InVitro Cabinet, Coy Laboratory Products). MMP activity in cell lysates was then analyzed using gel zymography. Protein samples (10 μL each; 6 μg per lane) were mixed in Novex Tris-Glycine Sodium Dodecyl Sulfate (SDS) Sample Buffer (Thermo Fisher Scientific) and then loaded onto Novex 10% Zymogram (Gelatin) protein gel for separation. Gel was electrophoresed at 126 V for 90 minutes. After electrophoresis, the gels were renatured in Zymogram renaturing buffer (Thermo Fisher Scientific) at room temperature for 30 minutes and incubated overnight at 37°C in Zymogram developing buffer (Thermo Fisher Scientific). The gels were stained with Coomassie blue staining solution (0.1% Coomassie R250 in 40% ethanol, 10% acetic acid) for 2 hours and then destained twice for 30 minutes in destaining solution (10% ethanol and 7.5% acetic acid). The presence of a clear band on a dark background indicated MMP activity; images were captured as described above.

### Human samples and scRNA-Seq analysis

Blood was obtained from healthy donors and patients with PAH and ILD (with and without PH) at the University of Florida Shands Hospital Pulmonary Hypertension and Interstitial Lung Disease Clinics (Gainesville, Florida). Participants were either healthy individuals (donor) or patients diagnosed with Group 1 idiopathic PAH, Group 3 ILD-associated PH, or ILD without associated PH. PH was diagnosed by right heart catheterization. Exclusion criteria included age less than 18 years and concomitant cardiopulmonary phenotype. Samples were then processed by a specialist into PBMCs and serum, then stored at –80°C until analysis.

PBMCs were subjected to flow cytometry for quantification of STING expression in various cell populations. Extracellular staining was performed with fluorochrome-conjugated surface antibodies for 30 minutes on ice, followed by Fixable Viability Dye (eBioscience 65-0865-14) for 30 minutes on ice. The cells were then fixed, permeabilized, and incubated with intracellular markers for 30 minutes on ice. Data were acquired using a BD FACSymphony A3 Cytometer (BD Biosciences) with 5 lasers and analyzed with FlowJo version 10 software. A list of antibodies used is provided in Supplemental Table 4.

Serum samples were subjected to MILLIPLEX Magnetic Bead Panel (EMD MilliporeSigma MCYTOMAG-70K) for quantification of different interleukins, chemokines, and growth factors, including eotaxin, G-CSF, GM-CSF, IFN-γ, IL-1α, IL-1β, IL-2, IL-3, IL-4, IL-5, IL-6, IL-7, IL-8, IL-9, IL-10, IL-12 (p40), IL-12 (p70), IL-13, IL-15, IL-17, IP-10, KC, LIF, LIX, MCP-1, M-CSF, MIG, MIP-1α, MIP-1β, MIP-2, RANTES, TNF-α, and VEGF.

Patient and healthy donor control lung samples for generation of immunofluorescence images were obtained from the Lung Tissue Research Consortium as previously reported (31) (NIH BioLINCC, HLB02342020a). Upon antigen retrieval, sections were incubated with the primary antibodies CD31 (R&D Systems AF3628; 1:300) or α-SMA (MilliporeSigma A2547; 1:300) and STING (Novus NBP2-24683; 1:200) for 1 hour at room temperature. Sections were then incubated with corresponding secondary antibodies, prior to imaging as described above. Of note, sections were mounted using Vectashield Vibrance Antifade DAPI (Vector Laboratories); thus, DAPI was used as a nuclear counterstain. Images were qualitatively interpreted to identify patterns of coexpression between STING and cell-specific antibodies.

scRNA-Seq data were obtained from the NCBI’s Gene Expression Omnibus database (GEO GSE210248). For the analysis, only samples from patients with PAH and healthy donors (Donor) were selected, specifically the following 6 samples: Donor_1, Donor_2, Donor_3, PAH_1, PAH_2, and PAH_3. Each sample was processed individually using the Seurat package (v5) (68). Quality control (QC) steps included removing cells with over 5% mitochondrial gene content, fewer than 200 detected features (genes), or an abnormally high number of features exceeding 3 times the median absolute deviation. After QC, highly variable genes were identified using the FindVariableFeatures() function with default parameters. Gene expression data were normalized, scaled, and centered using the ScaleData() function. Principal component analysis (PCA) was conducted on the variable genes, and the first 20 principal components were used to construct a shared nearest neighbor (SNN) graph. Clustering was performed using FindClusters() with a resolution parameter of 0.2. For visualization, dimensionality reduction was performed using Uniform Manifold Approximation and Projection (UMAP), based on the same 20 PCs. Cell type annotation was conducted using canonical marker genes, including *DCN*, *CD14*, *CXCR4*, *TAGLN*, *CCL5*, *S100A8*, *VWF*, *NKG7*, *BGN*, *TSPAN7*, *IGKC*, *CD1C*, *SFTPC*, and *TPSAB1* (25). Subsequently, genes encoding cGAS/MB21D1, STING1, type I interferon A and B, involved cGAS/STING pathway, and *IL6*, *CCL7* (*C6orf150*, *CCL5*, *CXCL10*, *IRF3*, *TBK1*, *TMEM173*, *STAT1*, *CCL2*, *CCL20*, *IFNA*, *IFNB*, *STAT6*, *DDX41*, *IFNAR1*, *IFNAR2*, *IL6*, and *CCL7*) were used for KEGG pathway enrichment analysis for both the PAH and Donor groups by ShinyGO 0.82 (69) (https://bioinformatics.sdstate.edu/go/). Genes for each KEGG pathway were retrieved using BioServices Python library (70). The expression levels of all genes within each pathway were summed to obtain a pathway-level expression level. For each cell type, differential expression analysis for each pathway between PAH and donor groups was then performed using Wilcoxon’s rank-sum test. *P* values were adjusted for multiple comparisons using the Benjamini-Hochberg (BH) method. Pathways were considered differentially regulated if the adjusted *P* value was ≤0.05 and the absolute log fold change (|FC|) was ≥1.5.

### Statistics

Statistical analysis was performed using GraphPad Prism 9.0 software. Quantitative data are presented as mean SEM. Each data point on bar graphs represents an individual mouse or human. Violin plots show mean, mode, and interquartile range of the data set. Data from each graph were collected across 1–3 individual experiment(s). Power analysis revealed 80% to more than 90% power to detect a change in the mean with *n* = 4. Therefore, all data sets were performed with *n* = 4–10. Data were pooled for biological replications performed in individual experiments for statistical analysis. Mean difference across groups was determined using ANOVA followed by unpaired 2-tailed Welch’s *t* test (unequal variance assumption), with Dunnett’s test used to compare each experimental group with a control group, accounting for multiple comparisons. For human cytokine data, Bonferroni’s method was used to correct for multiple comparisons, after 2-way ANOVA was utilized. A *P* value less than 0.05 was considered statistically significant.

### Study approval

Animal experiments and maintenance were approved by the IACUC of the University of Florida (protocol 08702). Animal studies are reported in compliance with Animal Research: Reporting of In Vivo Experiments (ARRIVE) and the Guide for Care and Use of Laboratory Animals (National Academies Press, 2011). Ethical approval of biobanking (human tissue collection) and data collection was received from the University of Florida Institutional Review Board (IRB201400744). Tissues were collected after written informed consent was obtained from donors.

### Data availability

Values for all data points in graphs are reported in the Supporting Data Values file.scRNA-Seq data were deposited in the NCBI’s Gene Expression Omnibus database (GEO 244864). R codes used for scRNA-Seq analysis are available in the GitHub depository (https://github.com/annt289/A-non-inteferon-dependent-role-of-STING-in-pulmonary-hypertension; commit ID 9be1c65). Additional data, analytical methods, and study materials that support the findings of this study are available from the corresponding author upon reasonable request.

## Author contributions

ATP and AJB wrote the manuscript, designed experiments, and performed data analysis. ATP, SV, ACO, CF, MDA, HP, JAC, BBL, KLL, RM, KER, PD, DL, and EE performed experiments. YZ, LC, and ZH analyzed scRNA-Seq data. ATP, MDA, HP, RP, and JAC performed histopathological analysis and scoring. LJ and HKQ provided support and advised on experimental design. AJB supervised the work.

## Supplementary Material

Supplemental data

Unedited blot and gel images

Supporting data values

## Version 1 05/22/2025

In-Press Preview

## Version 2 07/08/2025

Electronic publication
