# Supplementary material for: Opposing roles for myeloid and smooth muscle cell STING in pulmonary hypertension
Source: JCI Insight. 2025 May 22;10(13):e184792. doi: 10.1172/jci.insight.184792 (PMC12288902; doi:10.1172/jci.insight.184792)
Supplement: Supplemental data [file jciinsight-10-184792-s051.pdf]

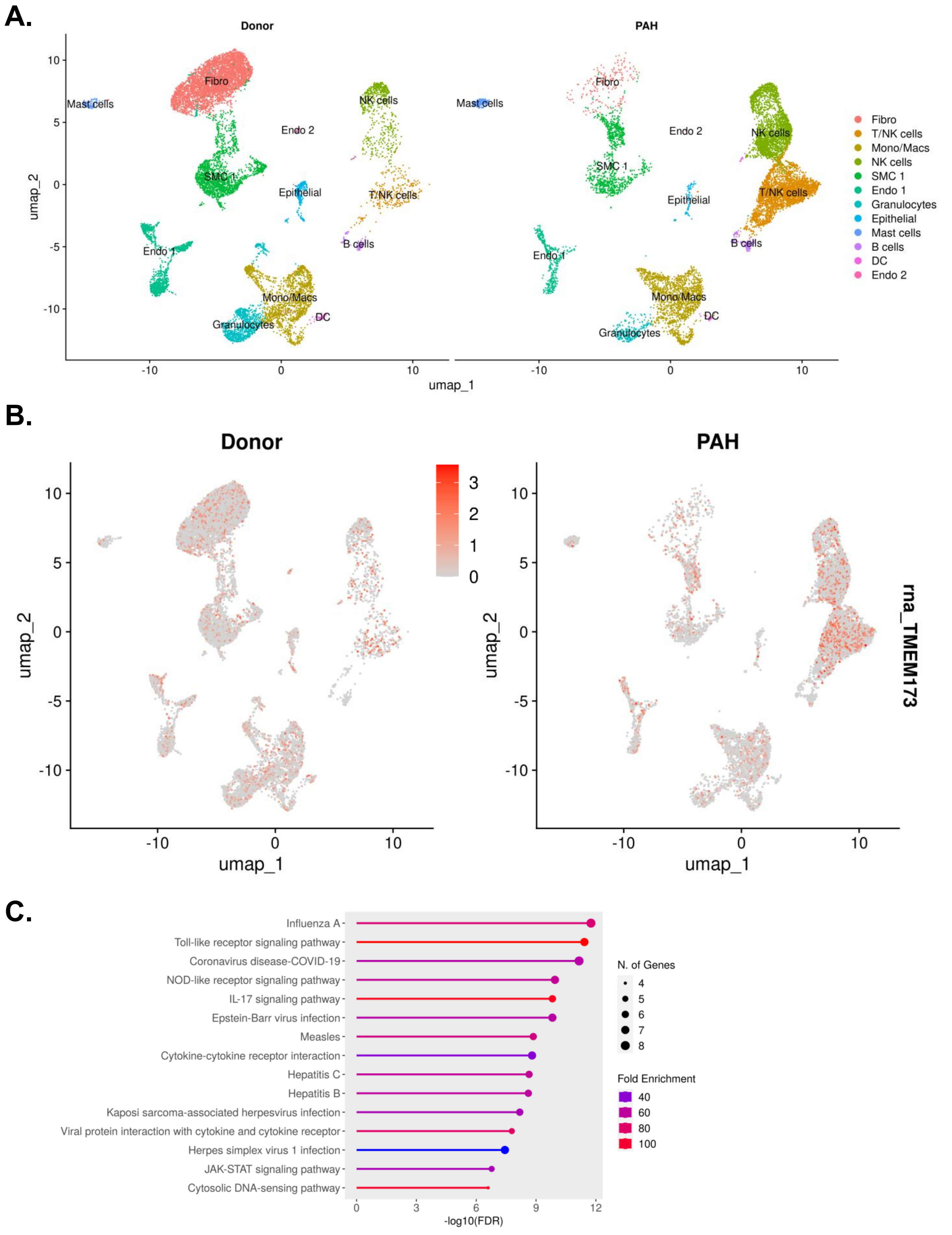

Figure S1. **Single-cell transcriptomics reveal differential expression of STING across multiple cell-types within the lungs of patients with pulmonary arterial hypertension (PAH).** (**A**) Uniform manifold approximation and projection (UMAP) of the lung vascular scRNA-Seq data of donor (n = 3) and PAH samples (n = 3) as previously reported by Crnkovic S., *et al. JCI Insight* (GSE210248). (**B**) Uniform manifold approximation and projection (UMAP) expression plots of TMEM173 (*Sting*), between donor and PAH group. (**C**) Gene Ontology (GO) analysis performed on cluster-enriched STING-relevant genes. Fisher's exact test with Benjamini-Hochberg adjustment, $P < 0.05$.

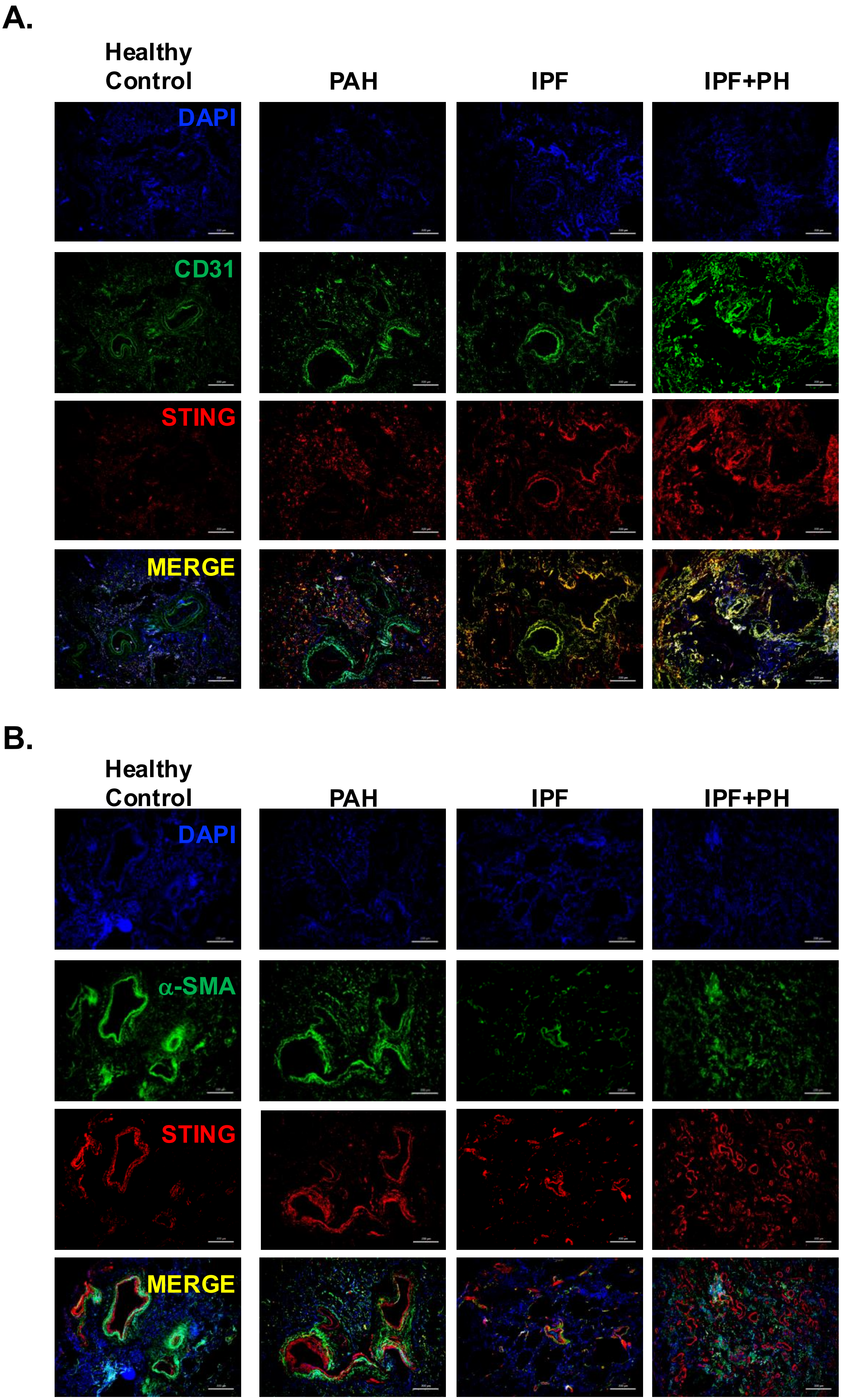

Figure S2. **STING co-expression in non-hematopoietic lineage cells within the lungs of patients with and without pulmonary hypertension (PH).** (**A**) STING (red) co-expression with pulmonary endothelial cells (CD31-expressing cells; green) in samples from either Healthy Controls or patients with pulmonary arterial hypertension (PAH), idiopathic pulmonary fibrosis (IPF) alone or with concurrent PH (IPF+PH) (**B**) STING (red) co-expression with pulmonary smooth muscle cells (α-smooth muscle actin [α-SMA]-expressing cells; green) in described patient cohorts. All images 10x magnification.

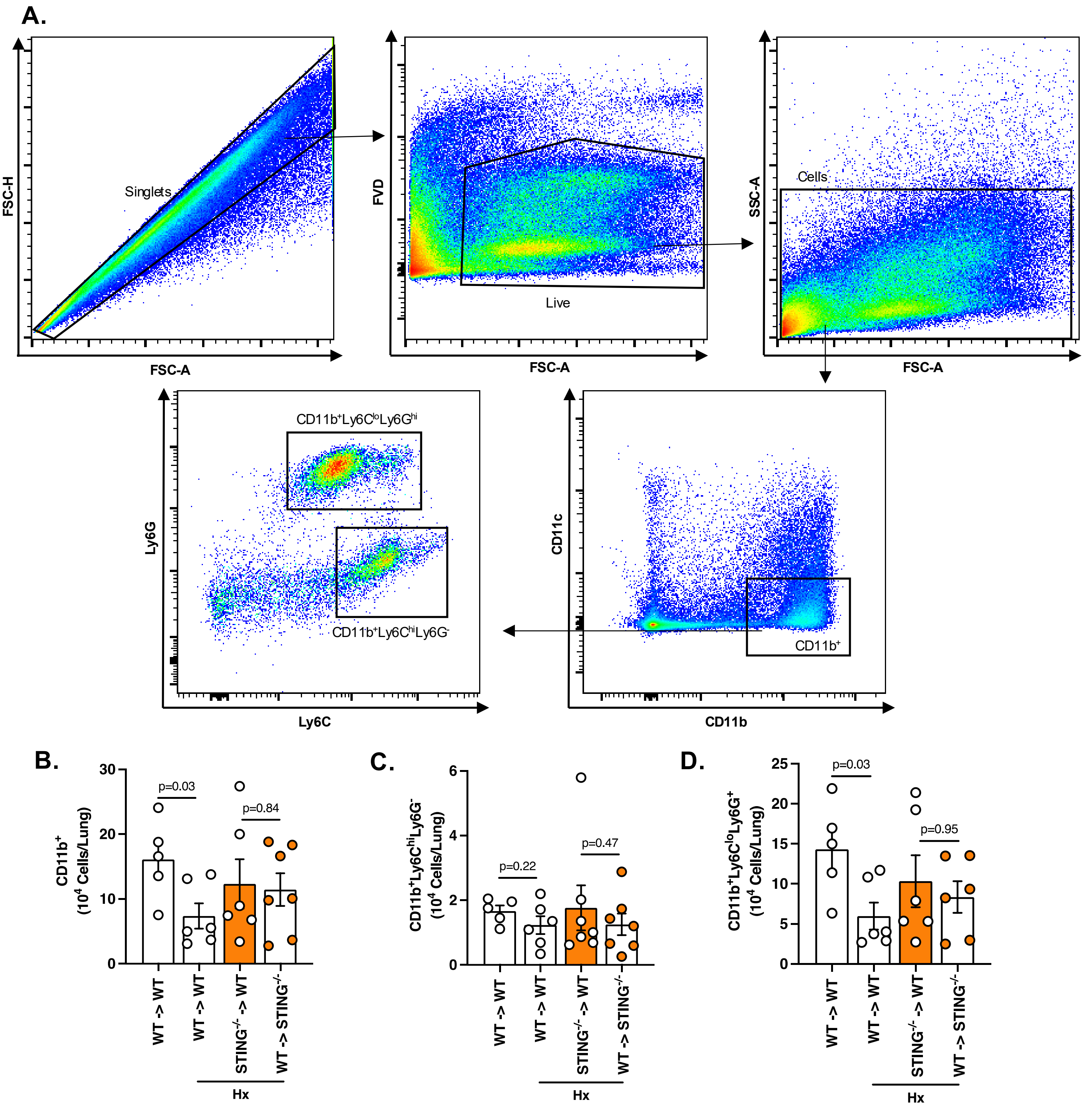

Figure S3. **STING expression on hematopoietic and non-hematopoietic cells play different roles in PH. (A)** Gating strategy for murine CD11b^{+}Cly6^{lo}Ly6G^{+} and CD11b^{+}Ly6C^{hi}Ly6G^{-} (**B – D**) Flow cytometric quantification of pulmonary infiltrated (**B**) CD11b^{+}, (**C**) CD11b^{+}Ly6C^{hi}Ly6G^{-}, and (**D**) CD11b^{+}Ly6C^{lo}Ly6G^{+} cells from designated mouse groups. Each dot represents an individual mouse (n=4-6/group). Column represents mean ± SEM. Significance level was calculated with an ANOVA followed by un-paired two-tailed Welch T's test corrected for multiple comparisons by use of Dunnett's test. P values are shown on graph. P<0.05 was considered significant.

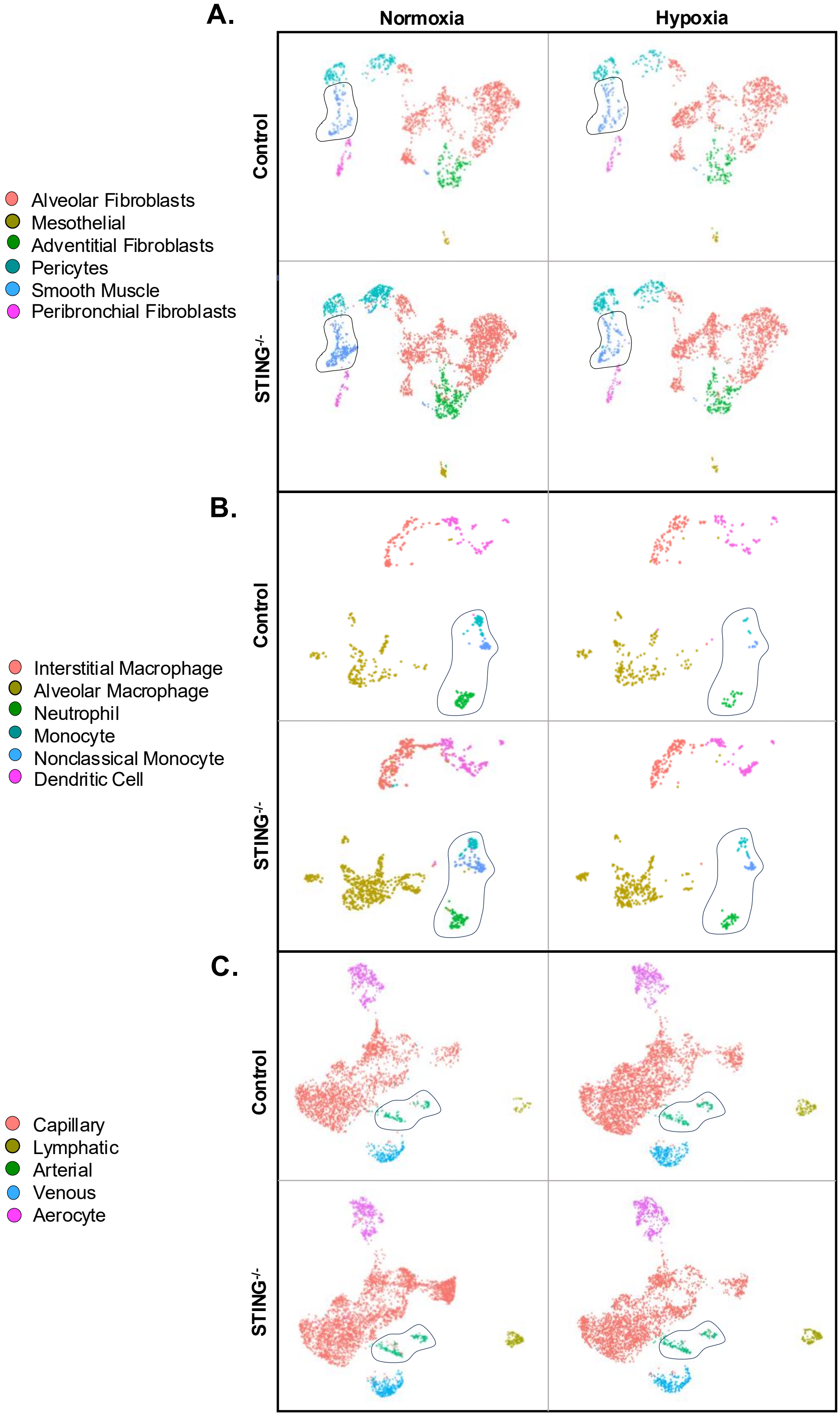

Figure S4. **scRNA-seq shows majority changes in stromal cells and myeloid cells in STING$^{-/-}$ mice exposed to chronic hypoxia.** UMAP projections of (**A**) Stromal cells, (**B**) Myeloid cells and (**C**) Endothelial cells of WT and STING$^{-/-}$ mice exposed to normoxia or chronic hypoxia (n=2/group).

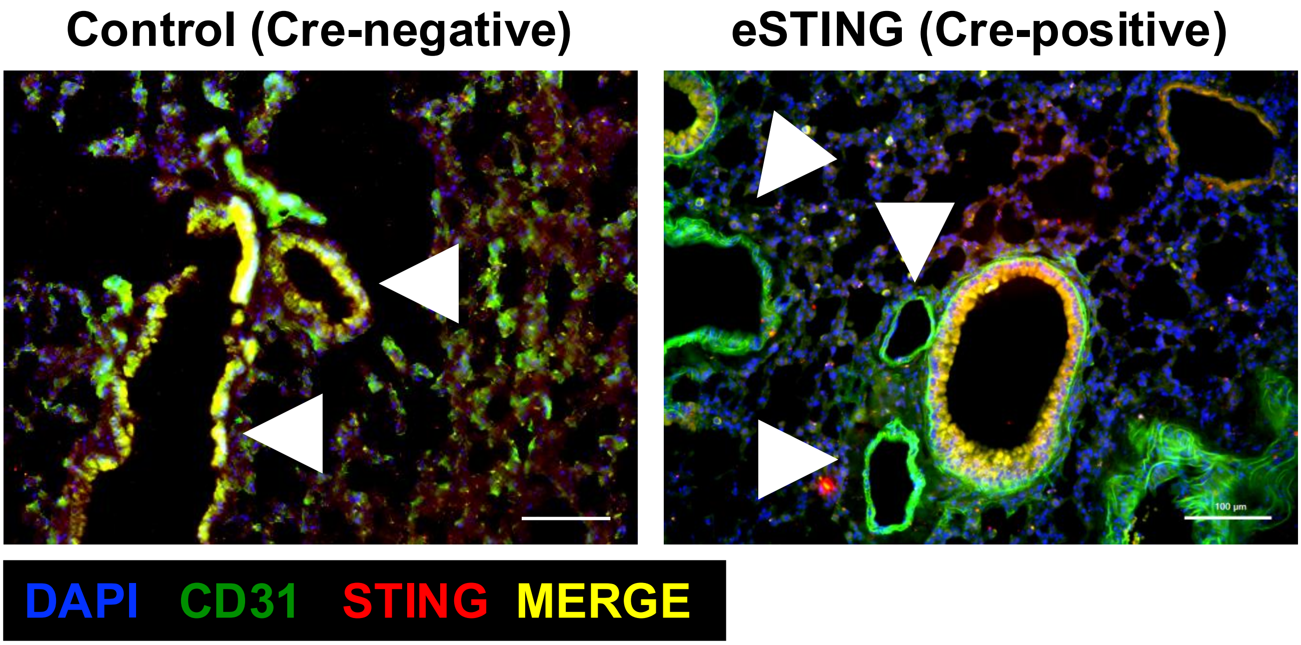

Figure S5. Lung tissue of mice demonstrating endothelial-specific deletion of STING in transgenic (eSTING) mice. Representative images showing immunofluorescence of lung tissues isolated from Control (VE-Cad.Cre-negative) and eSTING (VE-Cad.Cre-positive) mice stained for CD31 and STING. Pulmonary arteries are highlighted by white arrowheads, scale bar represents 100 μm. All images 20x magnification.

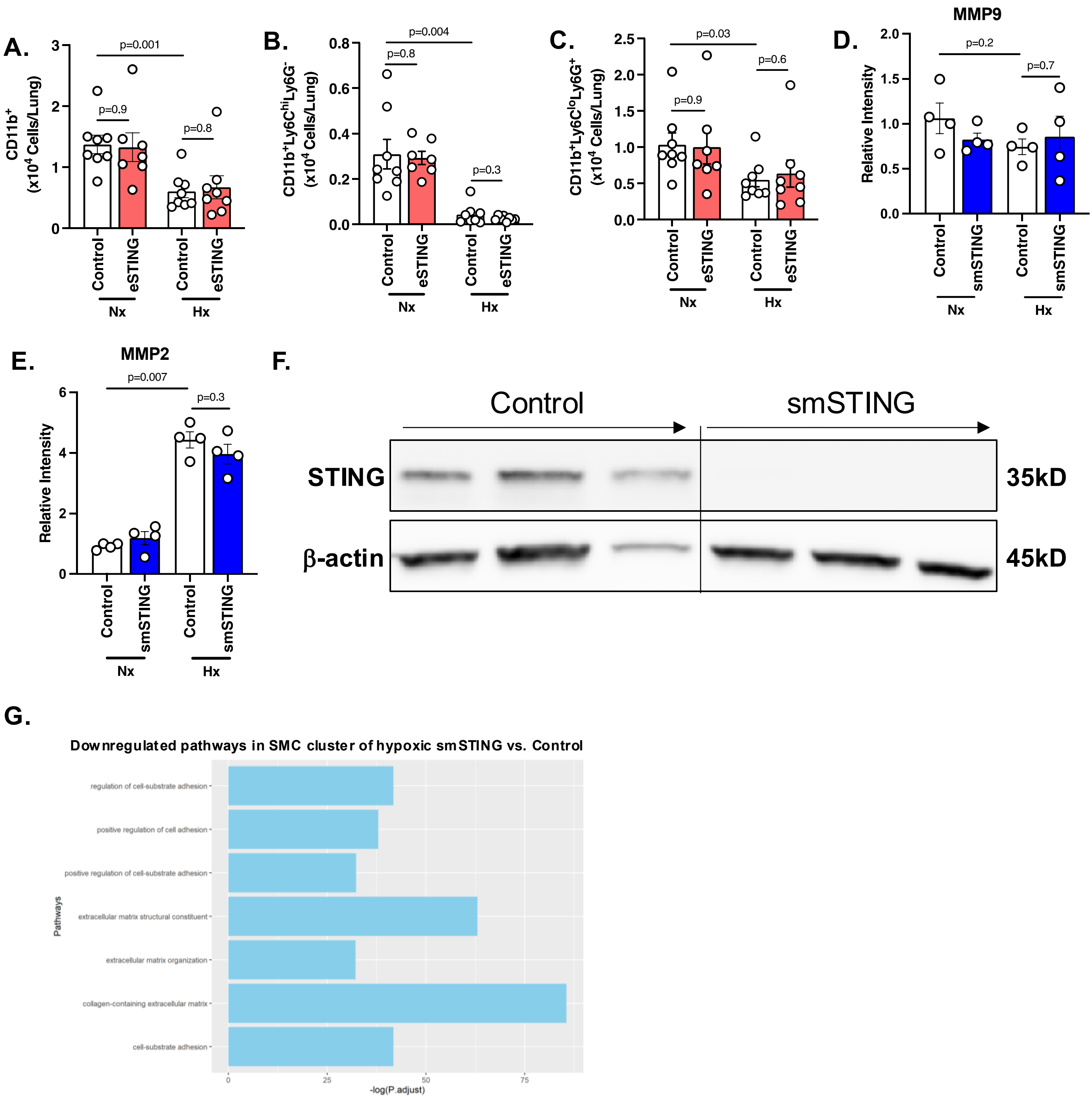

Figure S6: smSTING mice exposed to chronic hypoxia display an increase in pulmonary infiltrated proinflammatory cells. (**A – C**). Flow cytometric quantification of pulmonary infiltrated (**A**) CD11b$^+$, (**B**) CD11b$^+$Ly6C^{hi}Ly6G$^-$, and (**C**) CD11b$^+$Ly6C^{lo}Ly6G$^+$ cells from littermate control and eSTING mice from designated mouse groups. (**D and E**) Western blot quantification of (**D**) MMP9 and (**E**) MMP2 of littermate control and smSTING mice from different experimental groups. (**F**) Western blot confirming protein level deletion of smooth muscle cell STING-expression in smSTING cell versus Control cell isolates (n=3/grp; biologic replicates). (**G**) GO enrichment analysis of downregulated pathways in SMC of hypoxic smSTING lung compared to control. Column represents mean ± SEM. Significance level was calculated with an ANOVA followed by un-paired two-tailed Welch T's test corrected for multiple comparisons by use of Dunnett's test. P values are shown on graph. P<0.05 was considered significant.

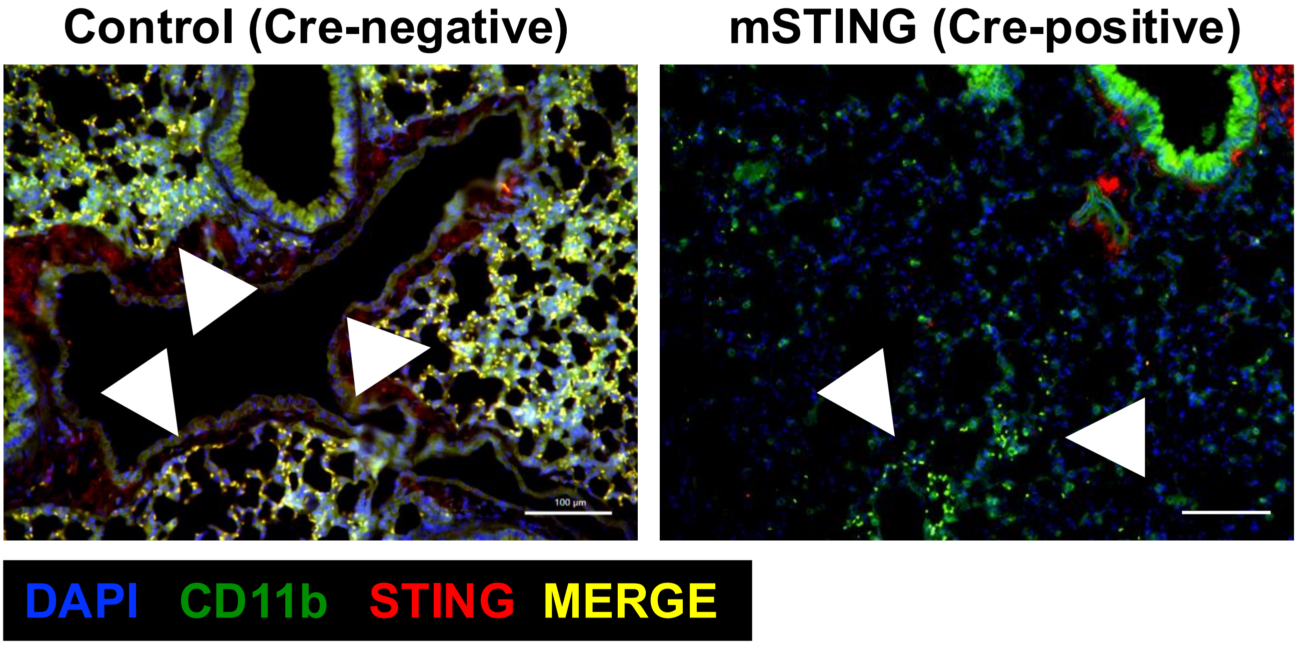

Figure S7. Lung tissue of mice demonstrating myeloid cell deletion of STING in transgenic (mSTING) mice. Representative images showing immunofluorescence of lung tissues isolated from Control (LysM.Cre-negative) and mSTING (LysM.Cre-positive) mice stained for CD11b and STING. Note absence of co-staining in bronchial epithelium of mSTING mice, consisten with LysM.Cre-model. Immune cell clusters are highlighted by white arrowheads, scale bar represents 100 μm. All images 20x magnification.

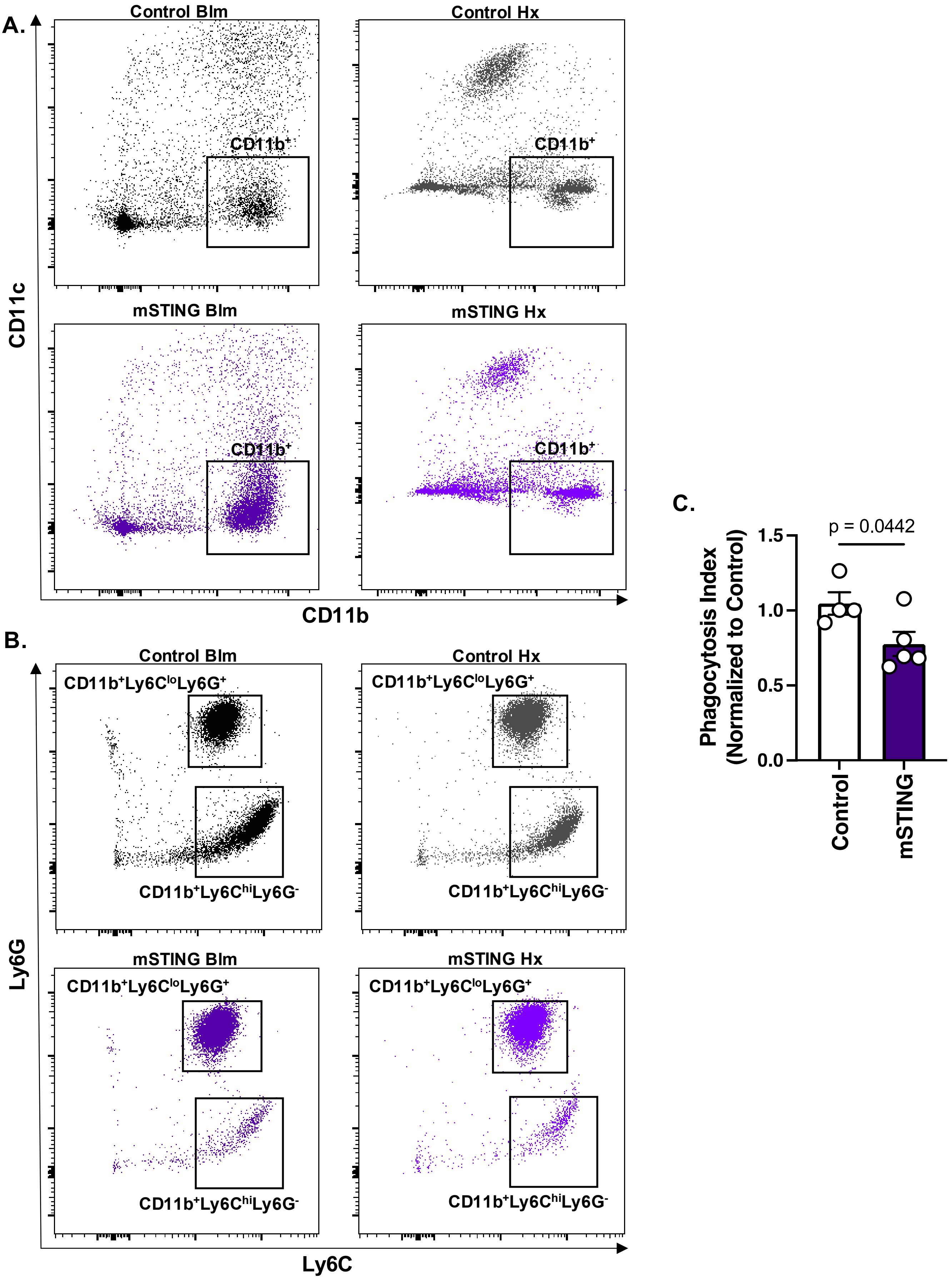

Figure S8: Accompanying figure detailing flow gating strategy for Figure 5, and phagocytosis index per genotype. Flow plot representation of (**A**) $CD11b^{+}$, (**B**) $CD11b^{+}Ly6C^{hi}Ly6G^{-}$ and $CD11b^{+}Ly6C^{lo}Ly6G^{+}$ cells from designated experiment groups. (**C**) Quantitative phagocytosis assay with labeled *E. coli* bioparticles. Column represents mean ± SEM. Significance level was calculated with un-paired two-tailed Welch T's test. P values are shown on graph. Biologic replicates displayed (n=4-5/group), with $P<0.05$ considered significant.

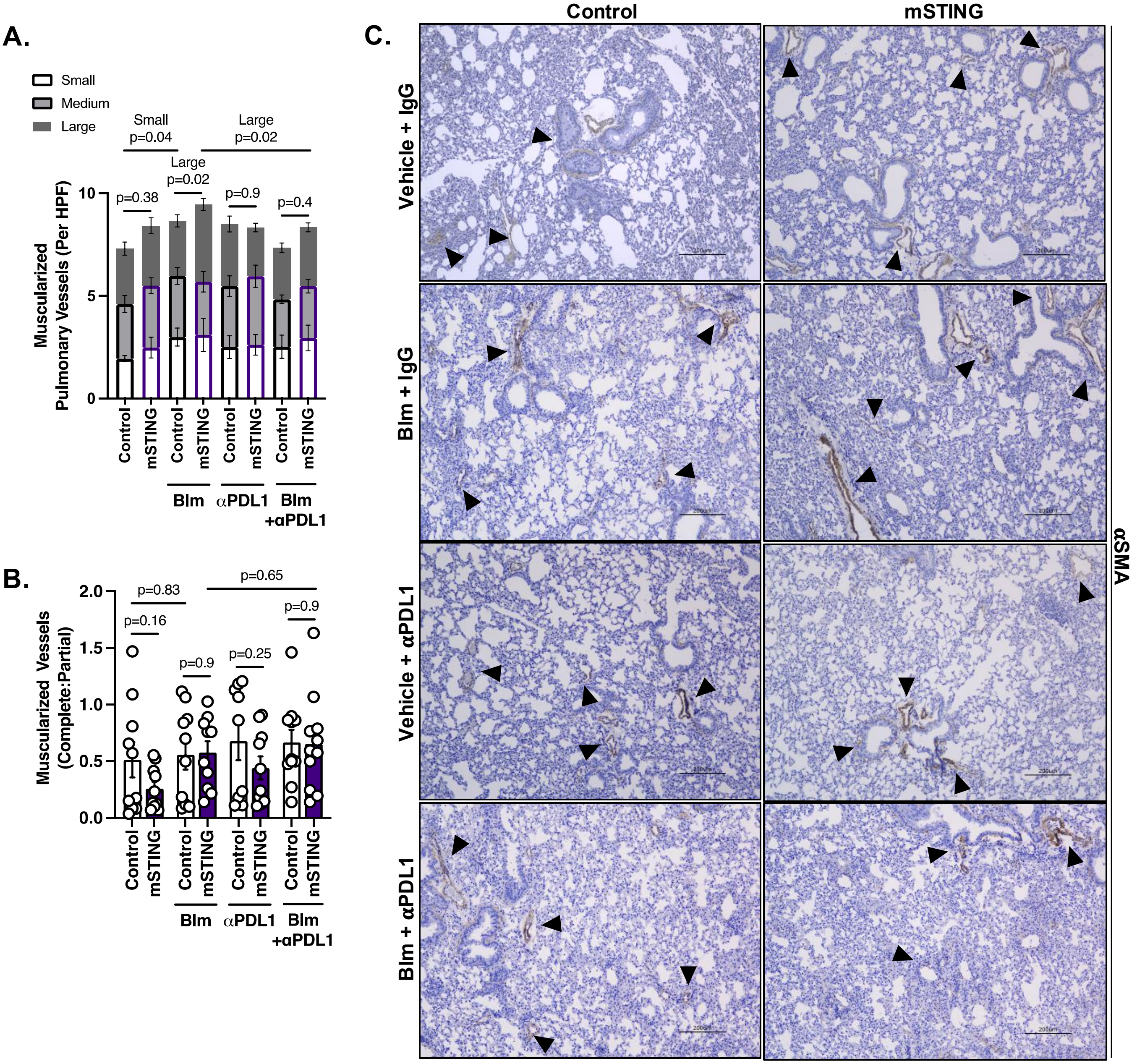

Figure S9: Anti-PD-L1 antibody abrogates severe PH in mSTING mice. (**A and B**) Quantification of (**A**) small, medium, large, and (**B**) complete or partial muscularized pulmonary vessels of WT and mSTING mice across designated experimental groups, assessing through αSMA IHC staining. (**C**) Representative images of αSMA (brown, arrowheads) IHC staining of formalin-fixed lung sections from mice across experimental groups. Scale bar = 200μm at 10x magnification. Each dot represents an individual mouse (n=5-10/group). Column represents mean $\pm$ SEM. Significance level was calculated with an ANOVA followed by un-paired two-tailed Welch T's test corrected for multiple comparisons by use of Dunnett's test. P values are shown on graph. P<0.05 was considered significant. P values from (**D**) were calculated from total vessels (small + medium + large) of mice from experimental groups, unless specified otherwise.

Supplemental Table 1: Demographic Factors and characteristics of PH patients						
**Characteristics**	**All Patients**	**Controls**	**PAH**	**PH-ILD**	**ILD**	**P-Value**
**Patients No (%)**	40 (100)	13 (32.5)	17 (42.5)	5 (12.5)	5 (12.5)	
**Age**						0.003
Mean +/- SEM	52.5 (3.08)	41.46 (5.29)	50.88 (4.30)	72.80 (2.63)	66.20 (5.76)	
**Gender**						0.066
Female	10 (62.5)	9 (69.2)	10 (58.8)	5 (100.0)	1(20.0)	
Male	6 (37.5)	4 (30.8)	7 (41.2)	0 (0.0)	4 (80.0)	
**Race and Ethnicity**						0.250
Caucasian	24 (60)	6 (46.2)	10 (58.8)	5 (100.0)	3 (60.0)	
Black	6 (15)	1 (7.7)	4 (23.5)	0 (0.0)	1 (20.0)	
Asian	4 (10)	4 (30.8)	0 (0.0)	0 (0.0)	0 (0.0)	
Hispanic	3 (7.5)	1 (7.7)	2 (11.8)	0 (0.0)	0 (0.0)	
Unknown	1 (2.5)	0 (0.0)	1 (5.9)	0 (0.0)	0 (0.0)	
Other	2 (5)	1 (7.7)	0 (0.0)	0 (0.0)	1 (20.0)	
**NYHA Functional Class**						NA
II	15 (37.5)	0 (NA)	11 (73.3)	1 (25)	3 (75)	
III	8 (20)	0 (NA)	4 (26.7)	3 (75)	1 (25)	

PAH; Pulmonary arterial hypertension, PH-ILD; Pulmonary hypertension related to Interstitial Lung disease, ILD; Interstitial Lung disease

Supplemental Table 2: Average chimerism percentage of WT and STING-/- chimeric mice in designated experimental groups		
Genotype	Exposure	Chimerism (%)
CD45.2 (WT) → CD45.1 (WT)	Normoxia	79.7
CD45.2 (WT) → CD45.1 (WT)	Hypoxia	72.5
$STING^{-/-}$ → CD45.1 (WT)		74.7
CD45.1 (WT) → $STING^{-/-}$		64.5

Supplementary Table 3. Primer sequences in mice

Gene	Primer	Sequence (5’ -> 3’)
LysM.Cre	Mutant	CCC AGA AAT GCC AGA TTA CG
	Common	CTT GGG CTG CCA GAA TTT CTC
	Wild type	TTA CAG TCG GCC AGG CTG AC
VeCad.Cre	Reverse	TGT CCT TGC TGA GTG ACA GTG GAA
	Forward	GCA GGC AGC TCA CAA AGG AAC AAT
SMA.Cre	Reverse	ACA TGT CCA TCA GGT TCT TGC
	Forward	GGT GTT AGT TGA GAA CTG TGG AG
$STING^{-/-}$	Forward	TTTTCATCTGCCTTCCAGGT
	Reserve	GCGCACACACACTAAAAACTG

Supplementary Table 4. FACS antibodies for flow cytometry used in human and mice.

Target	Supplier	Clone
**Mouse**		
CD11b ef450	Affymetrix	M1/70
CD11c PE-Cy7	Affymetrix	N418
Ly6C BV711	BioLegend	RB6-8C5
Ly6G BV605	BioLegend	1A8
PD-L1BV785	BioLegend	B7-H1
FVD APC-Cy7	eBioscience	
pIRF3 AF647	Cell Signaling Technology	D6O1M
CD3 PE-Cy7	Biolegend	17A2
CD4 BV605	Biolegend	GK1.5
CD8 BV785	Biolegend	53-6.7
CD25 FITC	Biolegend	PC61
PD-1 PE-CF594	BD Biosciences	J43
Foxp3 PE	Biolegend	MF-14
**Human**		
CD33 APC	BD Biosciences	WM53
HLA-DR FITC	BD Biosciences	L243
CD11b AF700	BD Biosciences	ICRF44
CD15 PE-Cy7	BD Biosciences	HIM1
CD14 Pacific Blue	BD Biosciences	M5E2
STING PE-CF594	BD Biosciences	RA3-6B2
