# Supplementary material for: Opposing roles for myeloid and smooth muscle cell STING in pulmonary hypertension
Source: JCI Insight. 2025 May 22;10(13):e184792. doi: 10.1172/jci.insight.184792 (PMC12288902; doi:10.1172/jci.insight.184792)

TIMP3 – Full unedited gel for Figure 3

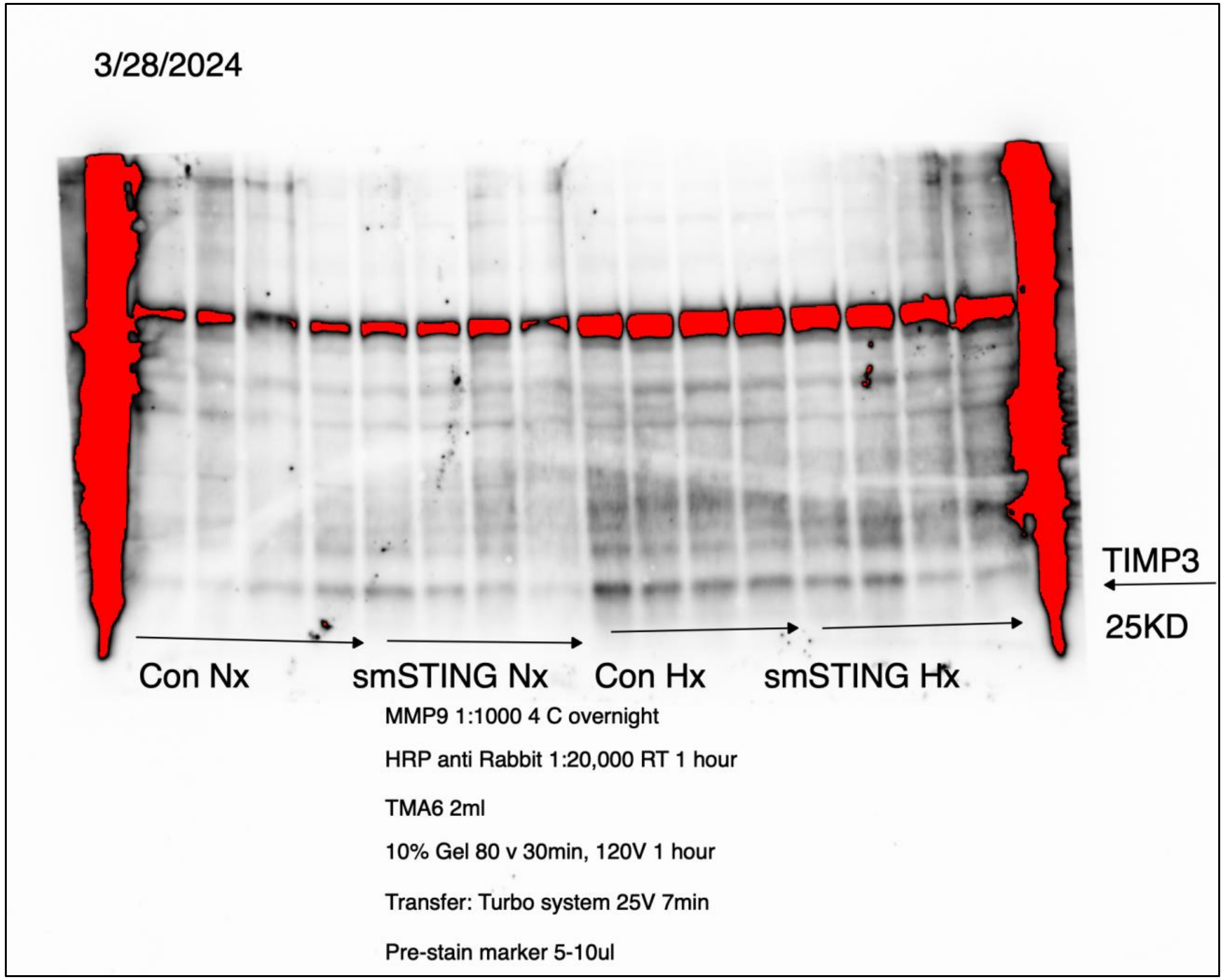

MMP8 – Full unedited gel for Figure 3

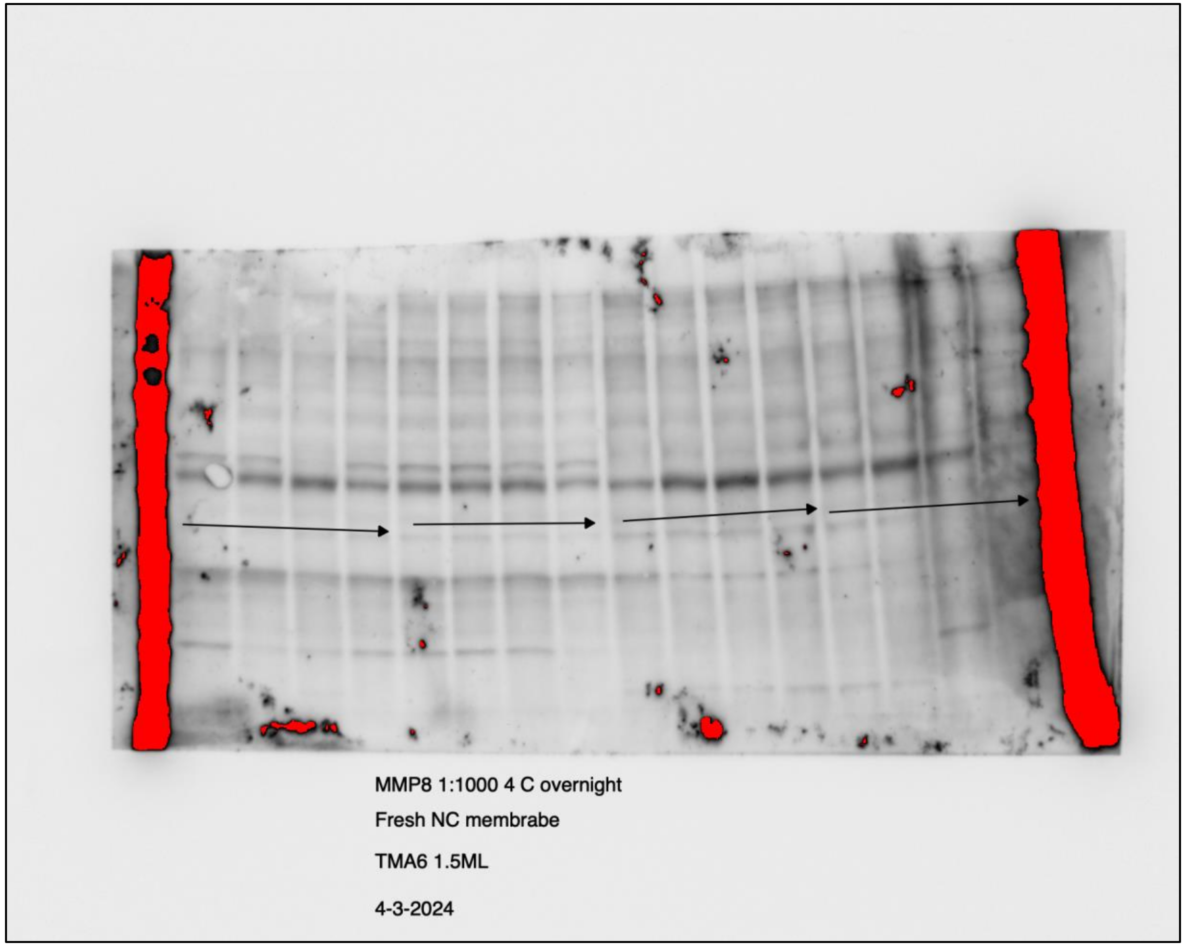

MMP2 – Full unedited gel for Figure 3

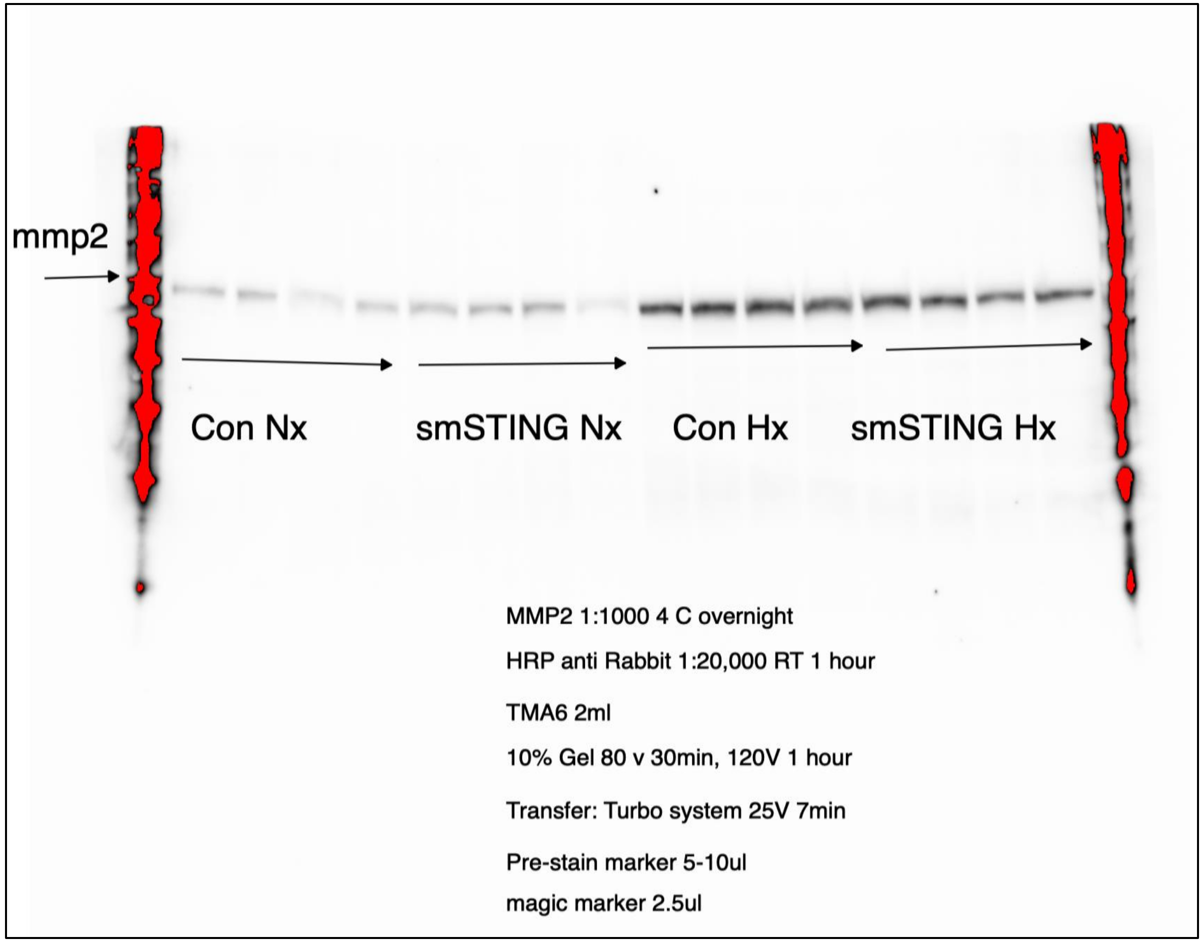

MMP9 – Full unedited gel for Figure 3

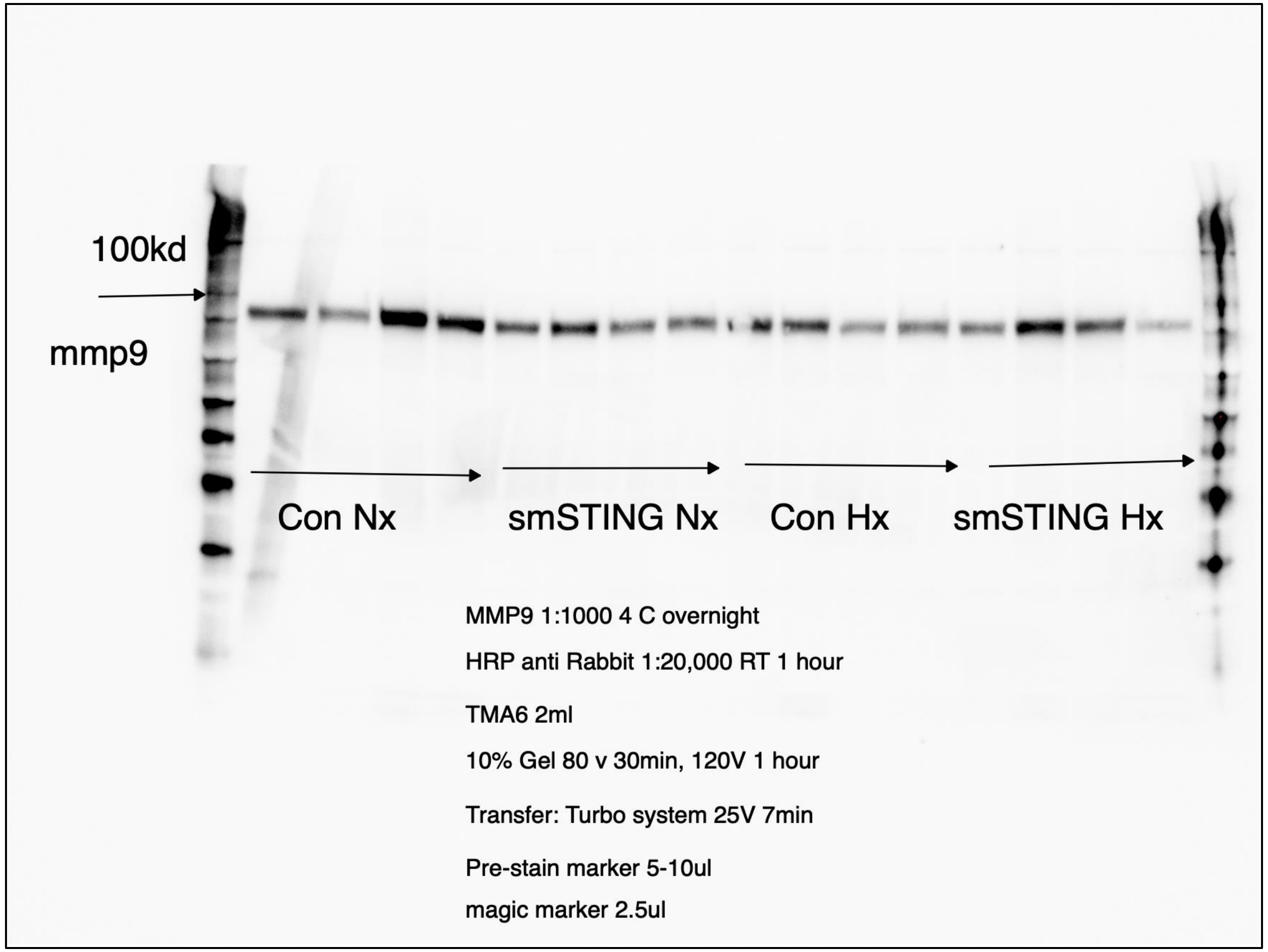

β-Actin – Full unedited gel for Figure 3

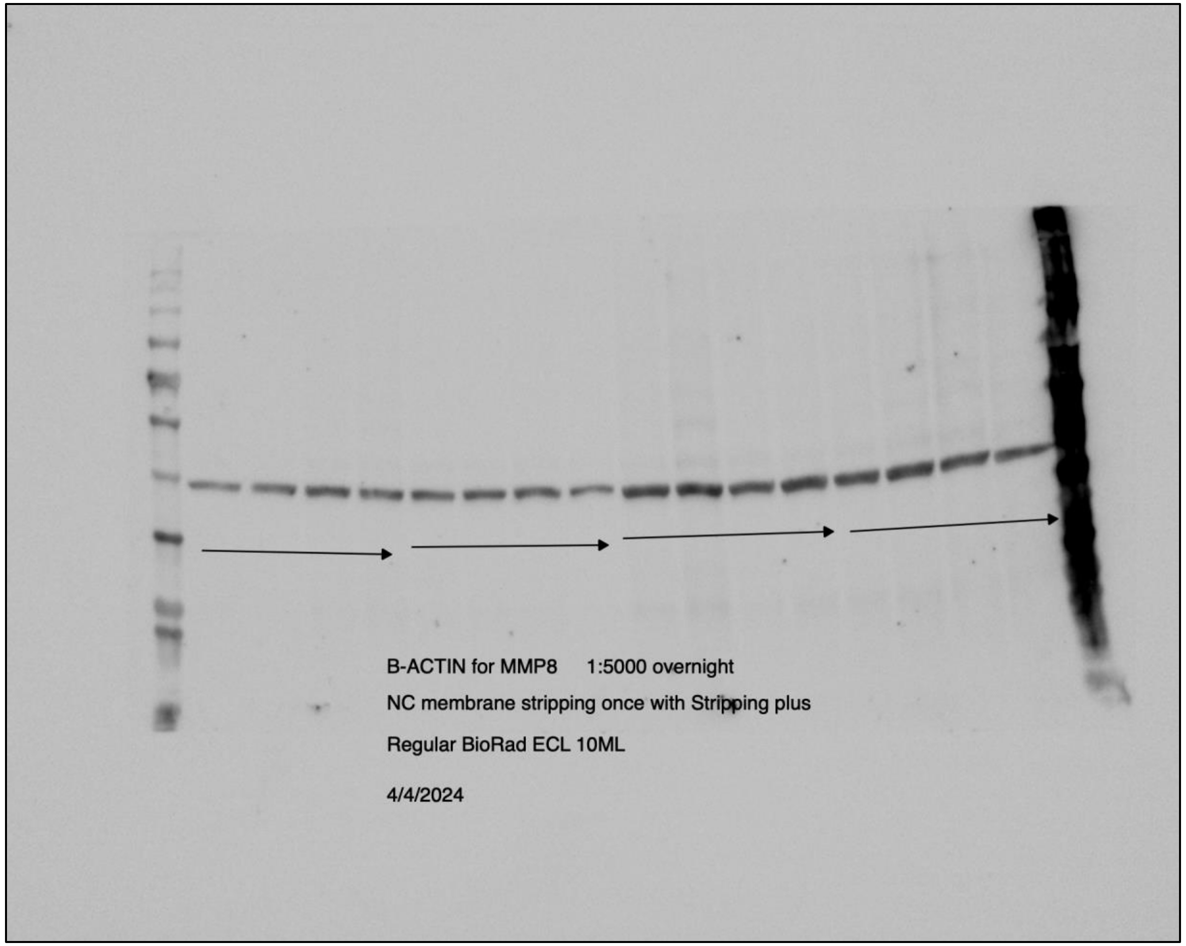

Gel zymogram – Full unedited gel for Figure 3

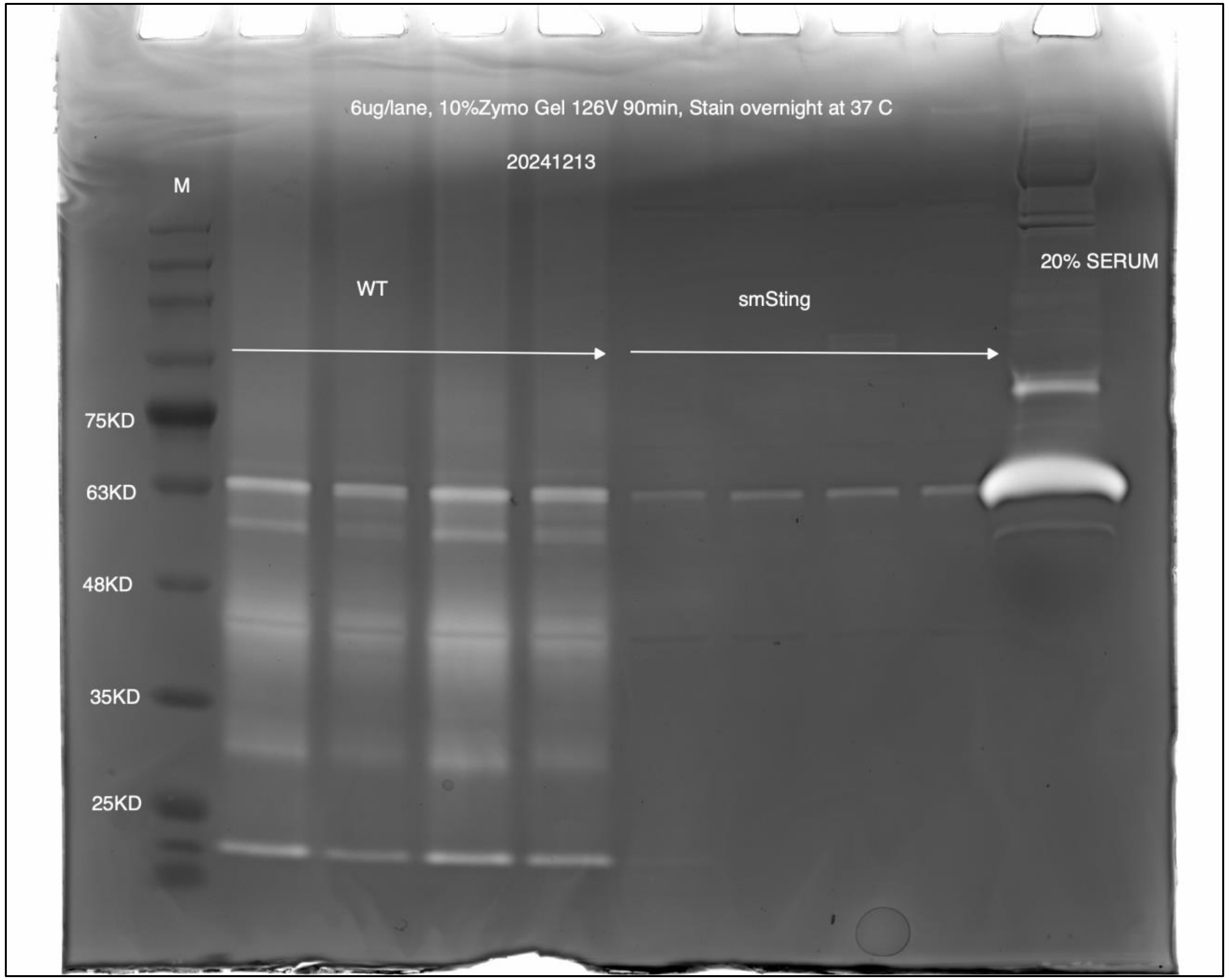

STING – Full unedited gel for Figure S5

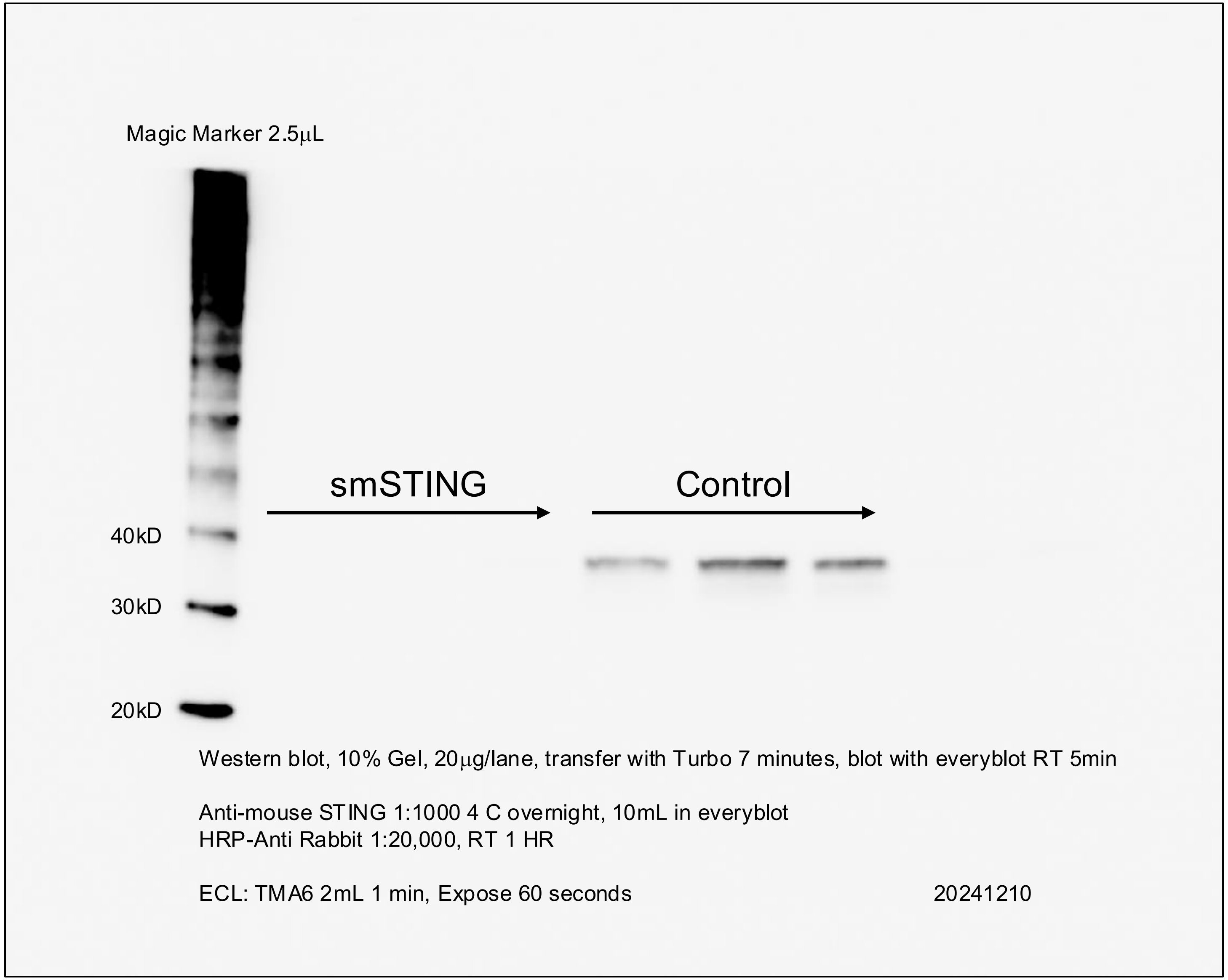

Supplement: Unedited blot and gel images [file jciinsight-10-184792-s052.pdf]
